# Therapeutic advantage of combinatorial chimeric antigen receptor T cell and chemotherapies

**DOI:** 10.1124/pharmrev.124.001070

**Published:** 2024-11-22

**Authors:** Meghan B. Ward, Amber B. Jones, Giedre Krenciute

**Affiliations:** Department of Bone Marrow Transplantation and Cellular Therapy, St. Jude Children’s Research Hospital, Memphis, Tennessee

## Abstract

Chimeric antigen receptor (CAR) T cell therapies have transformed outcomes for many patients with hematological malignancies. However, some patients do not respond to CAR T cell treatment, and adapting CAR T cells for treatment of solid and brain tumors has been met with many challenges, including a hostile tumor microenvironment and poor CAR T cell persistence. Thus, it is unlikely that CAR T cell therapy alone will be sufficient for consistent, complete tumor clearance across patients with cancer. Combinatorial therapies of CAR T cells and chemotherapeutics are a promising approach for overcoming this because chemotherapeutics could augment CAR T cells for improved antitumor activity or work in tandem with CAR T cells to clear tumors. Herein, we review efforts toward achieving successful CAR T cell and chemical drug combination therapies. We focus on combination therapies with approved chemotherapeutics because these will be more easily translated to the clinic but also review nonapproved chemotherapeutics and drug screens designed to reveal promising new CAR T cell and chemical drug combinations. Overall, this review highlights the promise of CAR T cell and chemotherapy combinations with a specific focus on how combinatorial therapy overcomes challenges faced by either monotherapy and supports the potential of this therapeutic strategy to improve outcomes for patients with cancer.

**Significance Statement:**

Improving currently available CAR T cell products via combinatorial therapy with chemotherapeutics has the potential to drastically expand the types of cancers and number of patients that could benefit from these therapies when neither alone has been sufficient to achieve tumor clearance. Herein, we provide a thorough review of the current efforts toward studying CAR T and chemotherapy combinatorial therapies and offer perspectives on optimal ways to identify new and effective combinations moving forward.

## Graphical abstract

**Figure:**
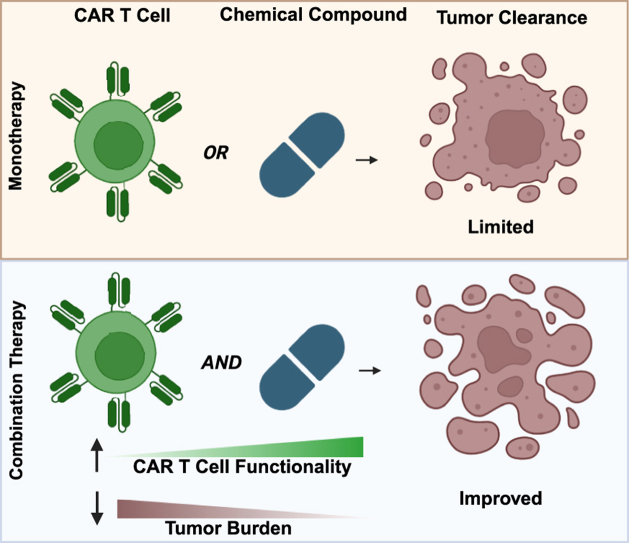

## I Introduction

Despite major advances in cancer research and treatment in just over 50 years since the signing of the National Cancer Act in 1971, cancer remains a significant healthcare crisis in the United States and worldwide. The American Cancer Society predicts that in 2024, >2 million Americans will be diagnosed with cancer, a record-breaking number. While cancer death rates are declining slightly, still >600,000 cancer-related deaths are predicted this year (Siegel et al, 2024). Moreover, the decline in the death rate is highly dependent on the cancer diagnosis. For example, significant progress in curing patients with lung and stomach cancer has been made in recent years, but prostate and central nervous system cancer death rates have not significantly improved (Siegel et al, 2023). Continued advancement in cancer treatment is needed to meet this growing health challenge.

Because molecular tools for understanding oncogenesis across cancer types have developed, targeted chemotherapeutics have been designed to tailor a treatment plan toward a specific diagnosis (Anand et al, 2023). However, some cancers have yet to see an effective targeted chemotherapy developed, or chemotherapeutics do not elicit curative responses on their own. Some of the major challenges faced by chemotherapeutics are lack of tissue specificity, limited half-life in the body, primary or acquired resistance, and significant short-term and long-term off-tumor toxicities (Chakraborty and Rahman, 2012; Anand et al, 2023). For example, primary or metastatic brain tumors are difficult to target owing to the lack of penetrance of chemotherapeutics across the blood-brain barrier (Upton et al, 2022; Terceiro et al, 2023). This severely limits the local concentration at the tumor site and renders chemotherapeutics ineffective. Chemotherapeutics often have short half-lives in the body (Lorscheider et al, 2021). Systemic delivery means that the drugs must be stable long enough to reach the site of the tumor and retain activity. However, this leads to an additional challenge—many healthy organs, tissues, and circulating cells are exposed to the active chemotherapeutic agents—leading to the myriad adverse side effects that many patients experience (Nurgali et al, 2018). Ideally, anticancer treatments will exhibit antitumor potency and be tissue penetrant, biologically stable, and minimize off-target effects.

In recent years, the number of annual US Food and Drug Administration (FDA) approvals of new cytotoxic chemotherapeutics has largely flatlined, while the approval rate of targeted biologics and immunotherapies has skyrocketed (Scott et al, 2023). Targeted biologics and immunotherapies have been shown to safely and specifically target tumor cells, limiting off-tumor toxicities and rendering them more tolerable than traditional chemotherapeutics (Schirrmacher, 2019; Waldman et al, 2020). The first immune-based therapy for cancer to gain FDA approval was the administration of interferon-alfa 2 (IFN-*α*2) to stimulate an anticancer response through both the innate and adaptive immune systems (Brassard et al, 2002; Eno, 2017). Since then, various monoclonal antibodies (mAbs), vaccines, and cell-based therapies have been approved as anticancer treatments (Twomey and Zhang, 2021). One mechanism for these therapies is to either prime (vaccines) or reinvigorate/boost (mAbs) the endogenous immune system to fight a tumor (Hargrave et al, 2023). Another is to isolate antitumorigenic immune effector cells from a patient, expand them ex vivo, and reinfuse them into the patient (Morotti et al, 2021). This strategy relies on the identification of an endogenous effector cell (T or natural killer) that has successfully infiltrated the tumor and has antitumor activity (Laskowski et al, 2022; Brummel et al, 2023). To broaden the applicability of the approach, researchers developed chimeric antigen receptors (CARs), synthetic molecules that recognize tumor-specific antigens and are transduced into peripherally isolated effector cells to elicit a targeted cytotoxic response (Eshhar et al, 1993; Feins et al, 2019; Albinger et al, 2021). This strategy does not rely on isolating endogenous tumor-reactive immune effector cells and is highly modular. Engineered T cell therapy is currently gaining the most traction with FDA approvals, and CAR T cell therapy is one of the primary focuses of this review.

## II Chimeric antigen receptor T cell therapy

**Fig. 1:**
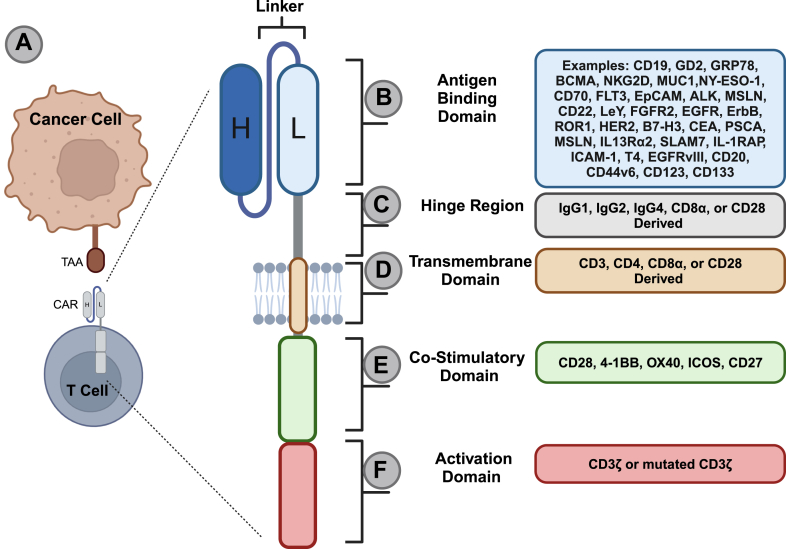
Structure of a second-generation chimeric antigen receptor. (A) CAR molecules on the surface of T cells recognize antigens on the surface of tumor cells and initiate CAR-mediated cytotoxicity. (B) The antigen binding domain is responsible for specifically recognizing the tumor antigen of interest. The blue box represents all antigens listed in Table 1 but is not an exhaustive list of all targetable antigens. (C) The hinge region imparts flexibility to the extracellular domain of the CAR molecule to facilitate antigen binding and downstream signal transduction. (D) The transmembrane domain anchors the CAR molecule into the T cell membrane. (E) The costimulatory domain provides additional support for CAR T cell persistence and viability. (F) The intracellular signaling domain is ultimately responsible for CAR-mediated cytotoxicity through the initiation of signaling cascades that release cytotoxic granules and cytokines into the tumor microenvironment.

CAR T cells are isolated T cells that are genetically engineered to express CARs. Briefly, the CAR molecule is loosely based on the natural cytotoxic T cell receptor (TCR) and recognizes tumor-specific antigens on the surface of tumor cells and transduces the recognition signal into activation of T cell cytotoxicity (Fig. 1A). CAR targets are identified based on high tumor-specific expression and low healthy tissue expression, limiting the potential of on-target off-tumor toxicity (Yee, 2014; Wang and Rivière, 2016). CAR T cell therapy has displayed promising success in treating certain hematologic malignancies, having received 6 approvals by the FDA (Kymriah, Yescarta, Tecartus, Breyanzi, Abecma, and Carvykti), and is actively being investigated as a treatment for additional blood-borne cancers, solid tumors, and brain tumors (Porter et al, 2011; Grupp et al, 2013; Kochenderfer et al, 2015; Asmamaw Dejenie et al, 2022).

Manufacturing CAR T cells begins by harvesting peripheral blood mononuclear cells from patients (clinically) or healthy donors (preclinically). T cells are isolated and activated, virally transduced to express the CAR of interest, and expanded for infusion or preclinical testing (Wang and Rivière, 2016). Structurally, CARs have 4 primary domains: the antigen recognition domain, hinge region, transmembrane domain, and signaling domain (Sadelain et al, 2013; Zhang et al, 2017a; Jayaraman et al, 2020). The antigen recognition domain (Fig. 1B) orients extracellularly and is responsible for CAR T cell recognition of the target antigen. Most CAR T cell constructs incorporate the single-chain variable fragment of a highly specific mAb for antigen recognition. Newer CAR designs have also used endogenous binding partners or peptide sequences known to strongly bind a target (Asmamaw Dejenie et al, 2022; Hebbar et al, 2022; Ibanez et al, 2023).

The hinge region (Fig. 1C) is a spacer between the antigen recognition and transmembrane domains. It facilitates antigen binding and signal transduction by imparting flexibility and adding length, making surface antigens more accessible and reducing rigidity and steric hindrance (Guest et al, 2005). Ultimately, the hinge domain has been found to regulate the threshold at which antigen can be recognized and downstream signaling is initiated (Fujiwara et al, 2020).

The transmembrane domain (Fig. 1D) is a crucial mediator for relaying extracellular antigen binding to intracellular signal transduction. It anchors the construct and offers essential stability for the CAR T cell. Selection of the transmembrane domain determines the overall surface expression level of the CAR construct as well as the recruitment of other signal-propagating components found in the native TCR (Fujiwara et al, 2020).

The signaling domain orients intracellularly and imparts the effector function of the CAR T cell upon antigen binding. The intracellular domain commonly consists of both costimulatory support (Fig. 1E) and cytotoxic signaling components (Fig. 1F) (Zhang et al, 2017a). Phosphorylation of the 3 immunoreceptor tyrosine-based activation motifs of CD3*ζ* transduces downstream cytotoxic signaling cascades in a standard CAR, similar to that of the TCR. CD3*ζ* phosphorylation ultimately results in the secretion of perforin, granzyme B, and other cytokines that induce apoptosis in targeted cells and inflammatory responses in the endogenous immune system. Costimulatory domains (Fig. 1E), such as CD28 or 4-1BB (CD137), which are currently included in all FDA-approved CAR T products, have been shown to augment the effector function of CAR T cells by increasing cytokine production, T cell proliferation, and potentiate antitumor activity over CAR T cells lacking costimulation (Finney et al, 2004). These costimulatory domains differ in benefits; CD28 promotes initial rapid tumor clearance, while 41BB may favor long-term persistence (Cappell and Kochenderfer, 2021). Other costimulatory domains are actively being investigated including OX40 (CD134), inducible T cell costimulator, and CD27 (Finney et al, 1998; Abate-Daga and Davila, 2016).

Multiple iterations of CAR structures have been investigated for therapeutic efficacy, leading to multiple generations of CAR design. All CAR T cell generations include the antigen recognition, hinge, transmembrane, and intracellular signaling domains. The first-generation CAR consisted of only a singular signaling domain with no costimulatory support. This single activation mechanism had limited cytokine production and poor overall effector performance. Second-generation CARs introduced a single costimulatory domain to the construct. All currently FDA-approved CAR T cell therapies are derived from second-generation CARs.

It was hypothesized that multiple costimulatory domains could provide further improvement to CAR T cell therapy, so the third-generation CAR incorporates ≥2 costimulatory domains. Unfortunately, the third-generation CAR failed to significantly outperform the second generation CAR (Morgan et al, 2010; Till et al, 2012), so all further iterations have been modified from the second-generation structure. The fourth-generation CAR includes the addition of an inducible or constitutive transgenic protein or cytokine to support T cell effector function by favoring T cell maintenance and survival. Some examples of cytokines expressed in fourth-generation CARs include but are not limited to interleukin (IL)-15, IL-2, and IL-7, which are necessary for cytokine activation and expansion (Chmielewski and Abken, 2015; Krenciute et al, 2017). A more recent example of a fourth-generation CAR design is the constitutive overexpression of 41BBL on the surface of CAR T cells (Zhao et al, 2015). 41BBL binds the 41BB receptor in *cis* and provides additional CAR T cell sustainability support (Nguyen et al, 2020).

As illustrated, CARs can differ substantially in structural design, spanning from the antigen being targeted to the intricate network of the signaling domain. CAR design may influence the efficacy at which tumors are targeted and is an important consideration as the field expands to combinatorial approaches. Taking this into consideration, throughout this review, we highlight the antigen being targeted as well as the costimulatory domain(s) expressed (denoted as Target.Costim).

**Fig. 2:**
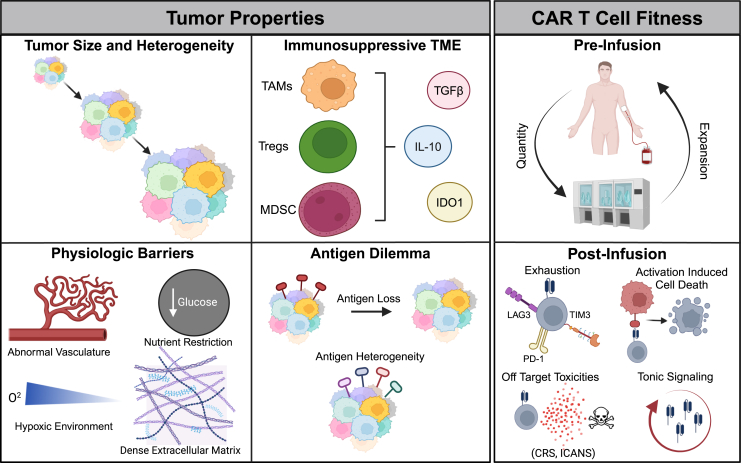
Challenges faced by CAR T cell monotherapy. Successfully implementing CAR T cells for treatment is hindered by both tumor and CAR T cell-specific limitations. Large, heterogeneous, and physically complex solid tumors are often difficult to fully eradicate by singular monotherapies. Coupled with downregulated or heterogenous antigen expression, CAR T cell homing and infiltration are limited. The tumor-associated immunosuppressive microenvironment also negatively affects the effector function of CAR T cells. Functionally, CAR T cells’ effectiveness can be hindered by poor preinfusion and postinfusion expansion. Premature exhaustion, off-target toxicities, and activation-induced dysfunction contribute to the incomplete antitumor potential of CAR T cells.

Collectively, there is substantial preclinical and clinical evidence supporting CAR T cell immunotherapy for cancer treatment. However, there are significant challenges that have hindered the holistic treatment success of CAR T cells against cancer. Established challenges of CAR T cells include properties of the tumor itself (limited identification and sustainability of novel antigens, aggressive tumor burden, hostile immune microenvironment) and CAR T cell–intrinsic deficiencies (unsuccessful homing and tumor infiltration, minimal persistence over time; Fig. 2)

## III Addressing challenges of chimeric antigen receptor T cell therapy with combinatorial chemotherapies

The challenges faced by CAR T cell therapy have hindered the success of clinical trials and ultimately the number of FDA approvals, especially for solid and brain tumors for which no therapies have been approved to date. Many groups are addressing CAR T cell–intrinsic deficiencies by introducing secondary genetic modifications that improve CAR T cell efficacy. Some examples include genetic knockdown of negative regulators such as programmed cell death protein 1 (PD-1) (Rupp et al, 2017), cytotoxic T-lymphocyte-antigen 4 (CTLA4) (Zhang et al, 2019), and lymphocyte activation gene 3 (LAG3) (Zhang et al, 2017b), and negative epigenetic regulators such as DNA methyltransferase 3-*α* (DNMT3A) (Prinzing et al, 2021), ten-eleven translocation 2 (TET2) (Jain et al, 2023), and suppressor of variegation 3-9 homolog 1 (SUV39H1) (Jain et al, 2024; López-Cobo et al, 2024). An alternate strategy is the expression of positive signaling molecules such as C-Jun (Lynn et al, 2019) or runt-related transcription factor 3 (Tang et al, 2023; Zhu et al, 2023), or constitutively active cytokine receptors (Bell et al, 2023). Secondary modifications have had instances of improving CAR T cell fitness/persistence and tumor infiltration, but other tumor-driven challenges such as antigen dilemma/tumor heterogeneity, tumor burden, and the hostile tumor microenvironment are challenging to address using these approaches (Rafiq et al, 2020). Additionally, secondary genetic modifications require thorough preclinical efficacy and safety testing, which is time consuming and expensive, making it slow to implement in the clinic. However, adding an already FDA-approved chemotherapeutic agent to a cancer regimen in combination with CAR T cells is much more readily achieved. In addition to chemotherapies, multiple other avenues have been investigated to support the antitumor activity of CAR T cells. For example, investigators have sought to increase the persistence and potency of CAR T cells by combining them with supportive cytokines, IFNs, and antibodies. Although noteworthy, we have narrowed the scope of this review to studies focused on combination treatments with CAR T cells and chemotherapeutics.

To our knowledge, Table 1 represents a comprehensive list of all preclinical investigations combining CAR T cells with chemotherapy. Our thorough literature review/search found 132 unique preclinical CAR T cells and chemotherapeutic combination studies involving 67 different compounds (Table 1). Studies are grouped based on the CAR T cell limitation that was addressed with combinatorial therapy. For more information, Supplemental Table 1 further elaborates on the mechanisms/primary findings for each publication. In the remainder of this review, we highlight some, but not all, published studies that explore the ability of chemotherapeutics to be used in combination with CAR T cells across multiple cancer types with varying CAR T cell targets. We highlight examples in which each of the challenges faced by CAR T cells can be addressed by a combinatorial approach. We further provide mechanistic insight into the chemotherapeutic augmented CAR T cell efficacy and how it may be more broadly investigated.

**Table 1:** List of preclinical CAR T cell and chemotherapy combination studies

Drug		US FDA Approval Status	Drug Description	CAR Target	Cancer Model	PMID
**Single-action drugs (grouped by CAR T cell challenge addressed)**						
Antigen dilemma (AD)	All transretinoic acid	Yes	Vitamin A derived retinoid	BCMA	MM	36722406
				CD38	MM	36918219
	Azacitidine	Yes	Hypomethylating agent	CD70	AML	35452603
	Bryostatin-1	Yes	Modulate protein kinase C	CD22	Leukemias and lymphomas	31110075
				CD22	B cell acute lymphoblastic leukemia	35222407
	Cisplatin	Yes	Alkylating agent	CD133	Gastric	33635343
	Crenolanib	Yes	Kinase inhibitor	FLT3	AML	29472720
	Cyclophosphamide	Yes	Lymphodepleting	NKG2D ligands	Tumor free	26122933
	Decitabine (Dec)	Yes	Hypomethylating agent	CSPG4	Ovarian	36291817
				CD19	Lymphoblastoma	31372000
	Dec with chidamide	Yes	with HDAC inhibitor	nanobodyCD70	AML	36932256
	Gemcitabine	Yes	Antimetabolite	GRP78	Pancreatic	37897831
	Ingenol-3-angelate	Yes	Protein kinase C agonist	B7-H3	Osteosarcoma	38561833
	Lenalidomide	Yes	Immunomodulatory agent	MUC1	MM	35840578
	Lorlatinib	Yes	Broad kinase inhibitor	CD19, GD2, ALK	Leukemia, NB	38039964
Tumor burden (TB)	ABT-737	No	Bcl-2 inhibitor	CD19	B Cell leukemia	23788110
	Azacitidine	Yes	Hypomethylating agent	CEA	Colorectal	30075754
	Celecoxib	Yes	COX2 inhibitor	CD19	B Cell leukemia	29904021
	Dabrafenib	Yes	MAPK inhibitor	GD2	Melanoma	25415284
	Decitabine	Yes	Hypomethylating agent	EGFR, CD44v6	Bladder	34868059
	Fluorouracil	Yes	Antimetabolite	CEA	Colorectal	30075754
	Indometacin	Yes	Nonsteroidal anti-inflammatory drug	CD19	B cell lymphoma	35882449
	Paclitaxel	Yes	Antimicrotubule agent	T4	Epithelial ovarian	30167862
	Rimiducid	Yes	Dimerizing agent	IL-1RAP	AML	33414517
	Sodium butyrate	No	HDAC inhibitor	CEA	Colorectal	30075754
	THZ1	No	Broad kinase inhibitor	EGFR	TNBC	33875483
	Trametinib	Yes	MEK inhibitor	GD2	Melanoma	25415284
	Vemurafenib	Yes	MAPK inhibitor	GD2	Melanoma	25415284
	Zanubrutinib	Yes	Src kinase inhibitor	CD19	Lymphoblastoma	36254554
Infiltration (I)	DMXAA	No	STING agonist	Neu	Breast	33382402
	Docetaxel	Yes	Antineoplastic agent	HER-2	NSCLC	30744691
	Rapamycin	Yes	mTOR inhibitor	EpCAM	AML	34233960
Immune microenvironment (IM)	All transretinoic acid	Yes	Vitamin A–derived retinoid	FGFR4	Rhabdomyosarcoma	35877472
	BLZ945	No	CSF1R inhibitor	B7-H3	Glioma	37971169
	Carboplatin	Yes	Alkylating agent	Lewis Y antigen (LeY)	Prostate	37660083
	Cyclophosphamide (Cy)	Yes	Lymphodepleting	CD19	Raji tumors	21487038
				CD19	Raji tumors	18477047
				CEA	Colon and breast	33796409
				PSCA	Prostate and pancreatic	33647456
	Cy with Fludarabine	Yes	With lymphodepleting	CD19	B cell leukemia	25940712
	Cy with Fludarabine	Yes	With lymphodepleting	B7-H3	Canine sarcoma	35405743
	Docetaxel	Yes	Antineoplastic agent	PSMA	Prostate	35962287
	Epacadostat	Yes	IDO1 inhibitor	FGFR4	Rhabdomyosarcoma	35877472
	Epacadostat	Yes	IDO-1 inhibitor	MSLN	ESCC	33828565
	L-NAME	No	iNOS inhibitor	FGFR4	Rhabdomyosarcoma	35877472
	Oxamate	No	LDHA inhibitor	EGFRvIII	GBM	37770937
	Pexidartinib	Yes	CSF1R inhibitor	FGFR4	Rhabdomyosarcoma	35877472
(IM)	PI-3065	No	PI3K inhibitor	ROR1, EGFRvIII	Breast	29760047
	SD-208	No	TGFβ inhibitor	FGFR4	Rhabdomyosarcoma	36722406
				ROR1	TNBC	32303620
	7DW8-5	No	Immunostimulant	ROR1, EGFRvIII	Breast	29760047
Fitness (F)	Acalabrutinib	Yes	Src kinase inhibitor	CD19	Lymphoblastoma	31899702
	AKT inhibitor VIII	No	PI3K inhibitor	CD19	B cell leukemia	29212954
	Carboplatin	Yes	Alkylating agent	ErbB	Epithelial ovarian	23898037
				EGFR	TNBC	35813488
	Celecoxib with aspirin	Yes	COX1/2 inhibitors	CD19	B Cell lymphoma	34122428
	Dasatinib (Dasa)	Yes	Src Kinase inhibitor	GD2	B Lymphoid leukemia	33795428
				GRP78	AML	35102167
				CD19	B Lymphoid leukemia	30814055
				CD19	Lymphoblastoma	31270272
				CD19	B Lymphoid leukemia	34289897
	Dasa with Ibrutinib	Yes	Src Kinase inhibitor	CD7	T cell leukemia	36086817
	Decitabine	Yes	Hypomethylating agent	CD19	Lymphoblastoma	33462245
				CD123	Leukemia	32973749
	Dexamethasone (Dex)	Yes	Anti-inflammatory synthetic glucocorticoid	IL13Ra2	GBM	35081104
				IL13Ra2	GBM	29103912
				CD19, CS-1, TAG-72	ALL, MM, ovarian	38140726
	Dex with methylprednisolone	Yes	Anti-inflammatory -synthetic glucocorticoid	CD19, MSLN	Leukemia	38475830
	Docetaxel	Yes	Antineoplastic agent	PSMA	Prostate	32728611
	Enasidenib	Yes	IDH2 inhibitor	CD19	Leukemia, osteosarcoma	38171332
	Ibrutinib	Yes	Src Kinase inhibitor	CD19	CLL	32683672
				CD19	Lymphoblastoma	32876369
				CD19	MCL	26819453
				CD19	CLL	26813675
				CD19	Lymphoblastoma	31899702
	Idelalisib	Yes	PI3K inhibitor	CD19	CLL	30737788
	IPI-145, CAL-101, or TGR-1202	No	PI3K inhibitor	MSLN	Melanoma	32383488
	JQ1	No	BET bromodomain inhibitor	EGFR	GBM	34058385
				CD19	CLL	34396987
	Lenalidomide	Yes	Immunomodulatory agent	CD133, HER2	GBM, breast	32967454
				CD19, BCMA	Lymphoblastoma	38123696
				NKG2D	Colorectal	37790973
				CD23	CLL	35259043
				CD19	Lymphoblastoma	33408186
				CD19	Lymphoblastic leukemia	33333026
				BCMA	MM	31395689
				CS1	MM	29061640
	LY294002	No	PI3K inhibitor	NKG2D	Breast, lung	35965586
				NKG2D	CML, pancreatic	30619300
				CD33	AML	29479065
	Metformin	Yes	Antihyperglycemic	CD19	Lymphoma	29662316
	Paclitaxel	Yes	Antimicrotubule agent	ICAM-1	Gastric	32995483
	Rapamycin	Yes	mTOR inhibitor	CD123, HER2,CD33	AML	32384544
				BCMA, CD123 (Natural killer cells)	AML	32384544
				IL-1RAP	AML	37173386
				CD19, BCMA	Lymphoma	31039141
				CD19	Lymphoblastoma	30890531
				CD19	Lymphoblastoma	29661681
	Regorafenib	Yes	Broad kinase inhibitor	EpCAM (NK Cell)	Colorectal	30410941
Fitness (F)	Rimiducid	Yes	Dimerizing agent	SLAMF7	MM	30740516
				CD123, HER2,CD33	AML	30740516
	Ruxolitinib	Yes	Janus kinase inhibitor	CD19	Lymphoblastoma	35101664
	SCH-58261	No	A2*α* receptor inhibitor	CD19	Leukemia	29720380
				MSLN	Ovarian	32151275
	Temozolomide	Yes	Alkylating agent	EGFRvIII	GBM	29872570
	THZ1	No	Broad kinase inhibitor	CD19	Lymphoma	33397398
	Trametinib	Yes	MEK inhibitor	GD2	NB	34382720
**Multifunctional drugs**						
AD, F	Azacitidine	Yes	Hypomethylating agent	CD123	AML	34750374
	Decitabine	Yes	Hypomethylating agent	NY-ESO-1	Breast, MM	26447882
AD, TB	S63845	No	Mcl-1 inhibitor	CD19	B cell malignancies	33362794
	Venetoclax	Yes	Bcl-2 inhibitor	CD19	B cell malignancies	33362794
				CD19	Multiple lymphoma models	35904479
F, I	Cisplatin	Yes	Alkylating agent	HER2	Lung	38282968
	Lenalidomide	Yes	Immunomodulatory agent	CD19	B cell lymphoma	37219767
				Wilms’ tumor 1	CML	34674611
				CD19, CD20	B cell non-Hodgkin lymphoma	27141398
				EGFRvIII	GBM	26450624
IM, TB	Sorafenib	Yes	Broad kinase inhibitor	GPC3	Hepatocellular	31078430
I, IM	Oxaliplatin (with cyclophosphamide)	Yes	Alkylating agent	ROR1	Lung	33357452
TB, F	Azacitidine	Yes	Hypomethylating agent	CD44v6	AML	37180104
	Decitabine	Yes	Hypomethylating agent	CD44v6	AML	37180104
	Eltanexor	No	XPO-1 inhibitor	CD19	Lymphoma, AML	34165175
	Ibrutinib	Yes	Src kinase inhibitor	CD19	Lymphoblastoma	36254554
	JQ1	No	BET bromodomain inhibitor	CD19, CD123	AML	36038554
	Linsitinib	Yes	IGF1R/IR inhibitor	GD2	Diffuse midline glioma	34964902
	Metformin	Yes	Antihyperglycemic mTOR inhibitor	CEA	Gastric	36827893
	Rapamycin	Yes	mTOR inhibitor	CD19	B cell lymphoma	21878902
	Selinexor	Yes	XPO-1 inhibitor	CD19	Lymphoma, AML	34165175
TB, I	JK184	No	Hedgehog inhibitor	B7-H3	Breast	36635683
AD, I, IM	Sunitinib	Yes	Broad kinase inhibitor	CAIX	Renal	31574023
F, I, TB	Metformin with rapamycin	Yes	mTOR inhibitor	EGFRvIII	GBM	38386420
F, I, IM	TY-52156, CAY10444	No	S1P3 receptor antagonist	EpCAM	Breast, colon	37591632

### A Antigen dilemma/tumor heterogeneity

When considering CAR T cell therapy for a given cancer, the first challenge is to identify a targetable tumor antigen (Wei et al, 2019). To be a viable target, the tumor antigen is ideally a surface-anchored protein that is highly expressed on tumor cells for sufficient CAR T cell activation, not expressed on surrounding or distal healthy tissues and not expressed on the CAR T cells themselves or any immune cells (Abbott et al, 2020; Breman et al, 2018; Wei et al, 2019). The expression on healthy tissues leads to on-target off-tumor toxicity that can be harmful to a patient (Castellarin et al, 2020; Flugel et al, 2023). The expression of CAR T cells leads to fratricide, or self-killing, which diminishes the potency of the therapy against cancer (Breman et al, 2018). Even once a target is identified, many tumors are heterogeneous, and not all cell populations may express the same surface antigen or at the same level, leading to incomplete tumor eradication. Different tumor types can regulate antigen expression in a variety of ways that may hinder CAR T cell efficacy. Broadly, the term “antigen dilemma” defines tumor-associated antigens that have low basal expression, are heterogeneous in nature, may downregulate in response to treatment, or are indiscriminate on tissue expression leading to off-target toxicities. To combat this limitation, it is desirable to identify chemotherapeutics that can modulate the expression of tumor antigens. Throughout this review, we will denote which branch of the antigen dilemma is being targeted by the different compounds. A complete list of compounds that address the antigen dilemma is found in Table 1 and Supplemental Table 1.

One way to accomplish this is to increase the expression of an antigen that is already present in tumor cells. Jetani et al (2018) published the first example of this with FMS-like tyrosine kinase 3 (FLT3).CD28 CAR T cells targeting acute myeloid leukemia (AML) (Jetani et al, 2018). FLT3.CD28 CAR T cell monotherapy had potent but limited antitumor efficacy against FLT3^+^ AML cell lines in vivo. Treatment with the preclinical FLT3 inhibitor crenolanib increased the surface expression of FLT3 on AML blasts and sensitized them further to FLT3.CD28 CAR T cell treatment. However, healthy hematopoietic stem cells (HSCs) express FLT3 and were found to be susceptible to elimination following FLT3.CD28 CAR T cell administration. Crenolanib did not increase FLT3 expression in HSCs, but all healthy HSCs were eliminated in vivo upon CAR treatment (Jetani et al, 2018). The safety of this strategy would require CAR T cell elimination after tumor clearance and re-engraftment of HSCs. HSC transplantation is very common for treating hematological malignancies in the clinic and would not present a significant roadblock to the clinical translation of this combinatorial approach (Hasan et al, 2024).

Similarly, anaplastic lymphoma kinase 1 (ALK1) has been identified as a promising immunotherapeutic target for neuroblastoma (NB) because it is upregulated on the surface of tumor cells but not on healthy tissues and has oncogenic pathology (Carpenter and Mossé, 2012; Wulf et al, 2021). Bergaggio et al (2023) developed the first human and murine ALK.CD28 CAR T cells in 2023 that effectively recognize and eliminate NB tumors highly expressing ALK. However, ALK.CD28 CAR T cells largely failed to elicit responses in preclinical models of low ALK-expressing cell lines. Previous literature has shown that treatment of NB with ALK inhibitors such as the FDA-approved agent lorlatinib has promising yet limited efficacy as a monotherapy (Goldsmith et al, 2023). However, one consequence of lorlatinib exposure is the stabilization of ALK surface expression, presenting a unique opportunity for combinatorial therapy with CAR T cells. In NB cell lines with low ALK expression, combinatorial therapy with lorlatinib and ALK.CD28 CAR T cells significantly extended survival over ALK.CD28 CAR T cell monotherapy in multiple preclinical models (Bergaggio et al, 2023). This is a promising strategy for NB because all patients with NB with high or low initial ALK expression could benefit from combinatorial treatment with lorlatinib through surface stabilization of the target antigen and innate antitumor activity of lorlatinib. ALK alterations and overexpression have also been shown in colorectal cancer and metastatic non–small cell lung carcinoma (Zhao et al, 2023), suggesting this combinatorial approach could be further explored in other cancer types.

A more challenging strategy is to consider inducing expression of a tumor antigen that is not naturally expressed on tumor cells. In a recent example, Harrer et al (2022) optimized a dosing regimen with the FDA-approved therapeutic decitabine and saw the expression of the CAR target chondroitin-sulfate proteoglycan 4 (CSPG4) on the surface of ovarian carcinoma cells that were negative before treatment. Treatment with chondroitin-sulfate proteoglycan 4.CD28 CAR T cells was effective only against decitabine-treated ovarian carcinoma cells, representing the first published chemotherapy-induced antigen expression with subsequent antigen-specific targeting. While promising, this approach was not tested in vivo and requires further preclinical optimization before considering clinical implications. Decitabine and structural variant azacitidine have also been shown to upregulate antigen expression of CD70, CD123, and CD19 in various hematological malignancies, highlighting the immense potential of this class of small molecules to modulate antigens in combinatorial CAR T cell therapy (El Khawanky et al, 2021; Leick et al, 2022).

### B Tumor burden

The next challenge for CAR T cell therapy to overcome is the burden (eg, size and aggressive growth) of target tumors. CAR T cell clinical trials most often enroll only patients who are relapsed or refractory to standard treatment and whose tumors are substantial in size and growth rate. An effective CAR T cell therapy needs to be able to tackle a large and aggressive tumor. While CAR T cells are effective killers, therapy benefits significantly from combinatorial approaches that can de-bulk tumors or slow their growth prior to or during CAR T cell administration. A complete list of compounds that address tumor burden is found in Table 1 and Supplemental Table 1.

A simple example of this approach would be to treat tumors with chemotherapeutics that have known antitumor efficacy along with CAR T cells so that both therapies are independently yet synergistically working to clear the tumor. A preclinical study by Zhang et al (2018b) in colorectal cancer models evaluated the efficacy of combinatorial efficacy of the kinase inhibitor regorafenib and epithelial cell adhesion molecule (EpCAM)-CAR natural killer (NK) cells. Regorafenib and EpCAM CAR-NKs had antitumor efficacy alone, but the combination significantly reduced the rate of tumor growth over either monotherapy in preclinical models. While not yet published preclinically, 1 phase 1 clinical trial has reported findings from 6 initial patients receiving glypican-3.41BB CAR T cells for the treatment of hepatocellular carcinoma (Fu et al, 2023). Of the 6, 4 patients received combinatorial treatment with sorafenib, a similar kinase inhibitor, during CAR T cell infusions. The data are not yet suggestive of an added or reduced benefit, but clinical testing of CAR T/kinase inhibitor combinatorial strategies will shed more light on the future viability of this combination.

In another example, chemotherapeutic treatment with the FDA-approved chemotherapy cisplatin is the first line of defense for gastric cancer. While cisplatin very effectively clears away more differentiated tumor cell populations, stem cell tumor populations are resistant to it and readily lead to tumor recurrence and metastasis. A published study has shown that these stem cells are positive for CD133 and that cisplatin treatment increases CD133 expression in stem cells (Lu et al, 2019). Han et al (2021) developed a CD133.CD28 CAR T product that was preclinically tested in combination with cisplatin treatment against gastric cancer. Compared with monotherapy, combination therapy resulted in decreased tumor burden in xenograft models, specifically with reduced stem cell populations in any remaining or relapsed tumor caused by CD133 CAR efficacy. This strategy highlights the ability of CAR T cells and chemotherapy to be mutually beneficial—cisplatin diminishes the tumor size, allowing CAR T cells to have less bulk to clear, and CAR T cells eliminate the tumor cells that are resistant to cisplatin treatment.

Another way to tackle aggressive tumor growth is to sensitize tumor cells to CAR T cell therapy, rendering it more effective. In one example, the pretreatment of leukemia models with indometacin sensitized cancer cells to CD19 CAR T cell therapy (Naval et al, 2019; Aboelella et al, 2022). Specifically, indometacin treatment increased the surface expression of death receptor 5 (DR5) on tumor cells. Tissue necrosis factor–related apoptosis-inducing ligand (TRAIL) is an endogenous ligand for DR5 that can be secreted by activated T cells to assist in tumor cell cytotoxicity (Naval et al, 2019). In this system, indometacin-induced DR5 expression on tumor cells sensitizes them further to CAR T cell cytotoxicity through both CAR recognition and TRAIL-mediated apoptosis. This strategy could be broadly applied to many cancers because the DR5/TRAIL signaling axis can be activated on all tumor cells.

### C Chimeric antigen receptor *T cell infiltration*

Successful trafficking of CAR T cells to tumor sites, especially solid tumors, can be significantly hindered by the physical and physiologic conditions of the tumor (Scharping et al, 2016). The restrictive nature of solid tumors to infiltrating lymphocytes is due in part to the fibrous, dense, and rigid nature of the extracellular matrix (ECM) (Salmon et al, 2012; Henke et al, 2019). Additionally, dysregulated tumor vasculature complicates the penetration and motility of CAR T cells to and through the tumor stroma (Lanitis et al, 2015; Park et al, 2023). Furthermore, many tumor types are characterized by a large degree of cellular heterogeneity that complicates successful tumor recognition and infiltration. Therefore, chemical compounds that can modulate tumor or CAR T cell properties to potentiate homing and infiltration are desirous. A complete list of compounds that address CAR T cell infiltration is found in Table 1 and Supplemental Table 1.

The DNA-damaging chemotherapy carboplatin has shown promising results in mitigating the dense ECM of solid tumors. In a recent study by Porter et al (2023), the administration of carboplatin increased the frequency of cancer-associated fibroblasts (CAFs) in a model of prostate cancer. First, CAFs underwent a proinflammatory shift, encouraging and assisting CAR T cell infiltration. Second, carboplatin-treated CAFs had increased expression of multiple ECM-degrading matrix metalloproteinase genes (*Mmp2, Mmp3, Mmp13, Mmp14, and Mmp27*). When treated in combination with carboplatin, Lewis Y antigen (Le^y^).CD28 CAR T cells had greater infiltration, accumulation, and cytotoxic potential against multiple prostate patient-derived xenografts (Porter et al, 2023). Other platinum-based therapies have also been shown to increase CAR T cell infiltration. Oxaliplatin was able to increase the infiltration and cytotoxic effects of receptor tyrosine kinase-like orphan receptor 1 (ROR1).41BB CAR T cells in a murine lung adenocarcinoma model. Oxaliplatin mediated immune landscape remodeling by inducing tumor-associated macrophages to express T cell–recruiting chemokines (*Cxcl9 and Cxcl10*). The resulting increased infiltration of ROR1.41BB CAR T cells served as a positive feedback loop as IFN gamma (IFN-*γ*) secretion by CAR T cells mediated more production of chemokines by the macrophages (Srivastava et al, 2021).

The hypoxic nature of tumors can further limit CAR T efficacy by diminishing CAR T cell fitness and tumor cell antigen expression, leading to decreased homing and recruitment potential (Sethumadhavan et al, 2017; Berahovich et al, 2019; Li et al, 2020). A 2020 study by Li et al (2020) demonstrated that combinatorial therapy of carbonic anhydrase IX (CAIX).41BB CAR T cells with sunitinib, an inhibitor of the receptor for tyrosine kinase, resulted in improved infiltration of CAR T cells into tumor tissue in a model of renal carcinoma. This was true under both normoxic and hypoxic conditions and was dose-dependent in relation to sunitinib. Sunitinib ultimately increased CAIX antigen expression on tumor cells, which allowed for better homing of CAR T cells to the tumor site. This showcases another way in which increasing antigen density on tumor cells promotes CAR T cell activity. Interestingly, improved infiltration caused by a CAIX.41BB CAR T cell combination with sunitinib in the renal carcinoma model did not produce a therapeutic benefit. However, there was a combinatorial therapeutic benefit in a metastatic lung model, illustrating heterogeneity in response, and that lack of response in 1 tumor model does not discount the potential utility of the approach in others. Instead, it suggests that infiltration was not the primary hindrance to CAR T cell efficacy in the renal carcinoma model. Of note, combined treatment of CAIX.41BB CAR T cells with sunitinib reduced the amount of immunosuppressive myeloid-derived suppressor cells (MDSCs) at the tumor site (Li et al, 2020). This is a common theme surrounding many of the compounds that increase the homing and infiltration of CAR T cells: they tend to also alter the tumor immune cell landscape either directly or indirectly.

### D Hostile tumor microenvironment

The immune system can significantly influence the efficacy of numerous therapies used to treat cancer, including immune and CAR T cell therapies (Johnson et al, 2022). For CAR T cells, hostility is largely driven by the immunosuppressive cells within the tumor that promote tumor immune escape during oncogenesis. The primary immunosuppressive cells include T regulatory (Treg) cells, MDSCs, and tumor-associated macrophages (TAMs) (Quail and Joyce, 2013). Tregs suppress T cell effector function and activation through secretion of immunosuppressive cytokines, competition for cytokines CAR T cells need to survive, and downregulation of stimulatory antigens on antigen-presenting cells (Thornton and Shevach, 1998; Wing et al, 2008). MDSCs have been shown to specifically inhibit CAR T cell function through a host of mechanisms, including Treg induction and stimulation, nutrient depletion, reactive oxygen species production, and anti-inflammatory cytokine secretion (Huang et al, 2006; Srivastava et al, 2010; Yu et al, 2013; Raber et al, 2014; Markowitz et al, 2017). TAMs contribute to immunosuppression by increasing the expression of amino acid–depleting enzymes such as indoleamine 2,3-dioxygenase (IDO-1), secreting immunosuppressive cytokines, and recruiting Treg cells (Yan et al, 2015, 2019; Ye et al, 2018; Wang et al, 2019). Of importance, TAMs are often the most abundant tumor-infiltrating immune cells, highlighting their importance in CAR T cell–based immunotherapy (He and Zhang, 2021; Bied et al, 2023). Chemotherapies that can mitigate immunosuppression in the context of CAR T cell therapy are extremely beneficial to the field, and many studies have illustrated the ability of chemotherapies to combat this challenge. A complete list of compounds that address the hostile tumor microenvironment is found in Table 1 and Supplemental Table 1.

A study led by Xia et al (2021a) used genomics and transcriptomics to reveal that the epigenetic modulator bromodomain-containing protein 4 mediates immunosuppression-driven resistance of glioblastoma (GBM) cell lines to epidermal growth factor receptor (EGFR).CD28.41BB CAR T cells. Treatment with the bromodomain and extraterminal motif inhibitor, JQ1, in combination with EGFR.CD28.41BB CAR T cells was able to suppress the induction of immunosuppressive proteins IL-6, IL-8, IDO-1, and programmed death ligand 1 (PD-L1). This combination was also able to extend the survival of the mice and prevent metastasis more significantly than either treatment alone (Xia et al, 2021a). CAR T therapy in GBM has also benefited from combined treatment with the lactate generation inhibitor oxamate to overcome the immunosuppressive microenvironment. Excess lactate production is a byproduct of aberrant glycolytic activity in tumor cells, which contributes to the increased expression of ATP-converting ectonucleotidases CD39 and CD73. CD39 and CD73 scavenge proinflammatory molecules to generate immunosuppressive byproducts. Oxamate treatment was shown to decrease the CD39 expression in TAMs and Treg cells, supporting a less hostile tumor microenvironment that allows for the higher antitumor activity of EGFRvIII.41BB CAR T cells (Sun et al, 2023).

Another compound investigated for immune-modulatory roles to enhance the efficacy of CAR T cells is the antineoplastic agent docetaxel. Docetaxel combination therapy was tested in a xenograft model of prostate cancer in combination with prostate-specific membrane antigen (PSMA) CAR T cells. The combination of docetaxel and PSMA.41BB CAR T cells was shown to decrease tumor burden and increase survival probabilities in vivo. Mechanistically, this combination ultimately reduced the ratio of immunosuppressive MDSCs to CAR T cells, providing a less immunosuppressive environment (Zhang et al, 2022).

As technology advances, there are now novel approaches to administer immune landscape-altering compounds. Shao et al (2021) revealed that nanosheets loaded with the IDO-1 inhibitor, epacadostat, supported antitumor activity CD19.CD28 CAR T cell therapy in esophageal squamous cell carcinoma. Epacadostat-loaded nanosheets reduced IDO-1 and facilitated production of the immunosuppressive metabolite kynurenine, supporting a more permissive tumor microenvironment for the CAR T cells. In vivo, tissue analysis revealed that mice treated with CD19.CD28 CAR T cells and epacadostat-loaded nanosheets had lower expression of exhaustion markers (PD-1 and T cell immunoglobulin and mucin domain-containing protein 3 [TIM-3]) and increased effector cytokines (IL-2, IFN-*γ*, and perforin) (Shao et al, 2021). In a study, Zhang et al (2018a) generated nanoparticles loaded with either the phosphatidylinositol-3 kinase inhibitor PI-3065 to regulate Treg cell subsets or 7DW8-5, a stimulatory agonist of effector cells. Using EGFRvIII.CD28 and ROR1.CD28 CAR T cells against breast cancer cell lines, the combination therapy with nanoparticles enhanced cytotoxicity and persistence of CAR T cells, supporting improved survival.

While targeting single populations within the tumor immune microenvironment has been beneficial, it is possible that other immunosuppressive mechanisms might compensate when one is eliminated, ultimately dampening the strength of a combinatorial approach. One unique way to holistically target the immunosuppressive compartment is to consider multiple immune-targeting compounds. A resulting “polypharmacy” approach was tested by Sullivan et al (2022) in a rhabdomyosarcoma model in combination with fibroblast growth factor receptor CAR T cells. The treatment schema involved fibroblast growth factor receptor CAR T cells along with antagonists to colony-stimulating factor-1 receptor, IDO-1, inducible nitric oxide synthase, tumor growth factor *β*, and PD-L1. The “polypharmacy” and CAR T cell combinatorial approach successfully controlled tumor burden and increased survival in an orthotopic model of rhabdomyosarcoma (Sullivan et al, 2022). While this is encouraging, the administration of several compounds comes with more potential for unwanted toxicities, and safety evaluation must be performed with extreme diligence.

One final important consideration for chemotherapy and CAR T cell combination therapy and the influence of the immunosuppressive microenvironment is the administration of lymphodepleting agents. Commonly, a combined regimen of cyclophosphamide and fludarabine is used as a lymphodepleting cocktail to promote a stable environment for CAR T cells to establish and persist in hematological malignancies and has been heavily reviewed elsewhere (Ramos et al, 2018; Amini et al, 2022; Lickefett et al, 2023). Some solid and brain tumor trials are also incorporating lymphodepletion prior to CAR T cell therapy as a way of removing immunosuppressive B and Treg cells from the tumor microenvironment (Heczey et al, 2017; Suryadevara et al, 2018). While lymphodepletion is often considered common practice, it is important to understand the interplay between lymphodepleting agents and lymphoid-based (CAR T) cell therapy. A clinical study led by Fabrizio et al (2022) illustrated the importance of determining optimal fludarabine concentrations in patients with B cell acute lymphoblastic leukemia undergoing CD19 CAR T cell therapy. Patients in this cohort treated with a suboptimal concentration of fludarabine had higher risks of relapse (Fabrizio et al, 2022). Therefore, because the field of CAR T cell therapy progresses, lymphodepleting schemas must be diligently designed to ensure that CAR T cells have the best conditions to initiate cytotoxic potential. Furthermore, the interactions of any combinatorial chemotherapeutics with lymphodepleting agents must be considered to devise appropriate treatment strategies that provide optimal therapeutic windows.

### E Chimeric antigen receptor T cell fitness

An additional challenge faced by all CAR T cell therapies is the limitation of antitumor activity caused by the inability of CAR T cells to persist in a functional effector state. Loss of functionality upon antigen exposure is called exhaustion and is marked by an increase in inhibitory receptors on the CAR T cell surface, such as PD-1 and CTLA4. mAb blockade of these signaling axes has been widely employed in the clinic (Wei et al, 2018) to reinvigorate the population of CAR T and endogenous T cells that have successfully infiltrated a tumor but have become exhausted (Makuku et al, 2021; Korman et al, 2022). Additionally, many secondary CAR T cell genetic alterations aim to improve the overall fitness (persistence, metabolism, effector function, and others) of tumor-infiltrating CAR T cells. To maintain fitness, CAR T cells need to be able to resist exhaustion (Chow et al, 2022), metabolically compete for limited nutrients in the tumor microenvironment (Peng et al, 2023), and form long-lasting memory subsets that can clear primary tumors and prevent relapse (Doan et al, 2024). There are many targetable pathways involved in these processes, as evidenced in Table 1 and Supplemental Table 1, in which the majority of CAR T cell and chemotherapy combinatorial studies have focused on CAR T cell fitness. Dosing may be accomplished in 2 fundamental ways. First, chemotherapeutics can be added to the CAR T cell manufacturing process to influence the pathways and properties of CAR T cells prior to infusion. Second, chemotherapies can be administered to patients during or after CAR T cell infusion to influence the fitness of CAR T cells within the tumor microenvironment. Here, we reviewed examples of both strategies.

#### 1 Chemotherapies added during the manufacturing process

Chemotherapies that can positively influence CAR T cells via addition in the manufacturing process are highly desirable because this approach can be broadly applied to any cancer therapy schema. A prime example of adding chemotherapeutics to the manufacturing process is the addition of the Src kinase inhibitor dasatinib. Dasatinib incubation keeps CAR T cells in a rested “off” state that enriches naïve or stem-like phenotypes prior to infusion (Mestermann et al, 2019; Weber et al, 2019, 2021; Watanabe et al, 2023). Evidence suggests that a high proportion of stem-like cells in CAR T cell infusion products may correlate with strong antitumor efficacy. Dasatinib is commonly used in clinical and preclinical CAR T cell manufacturing processes for this reason. Similarly, combination therapy of GD2.CD28.41BB with the insulin-like growth factor receptors/insulin receptor inhibitor linsitinib against diffuse intrinsic pontine glioma improved therapeutic efficacy by maintaining CAR T cells in a more undifferentiated central memory state (de Billy et al, 2021). This led to improved therapeutic efficacy at lower CAR T cell doses compared with CAR T cell treatment without linsitinib pretreatment. This result is very encouraging because diffuse intrinsic pontine gliomas are highly aggressive pediatric brain tumors that have no cure (Farrukh et al, 2023).

Previously, we discussed that the FDA-approved therapeutic decitabine can induce the surface expression of antigens on cancer cells and may address the CAR T cell antigen dilemma (Harrer et al, 2022). When added to the CAR T cell manufacturing process, decitabine alters the epigenetic landscape of CAR T cells to improve fitness by favoring memory formation and persistence (Wang et al, 2021). CD19.41BB CAR T cells pretreated with decitabine successfully eliminated large tumor burdens in preclinical models of acute lymphoblastic leukemia and prevented tumor growth upon rechallenge. This highlights the multifunctionality of a chemotherapeutic like decitabine: when administered to a tumor it can increase surface antigens, and when administered only to T cells it improves fitness.

CAR T cell fitness is commonly impaired by the metabolic hostility of the tumor microenvironment. Tumor cells and components of the tumor immune microenvironment outcompete CAR T cells for essential nutrients. In 2024, Si et al (2024) published a study evaluating the impact of pretreating CAR T cells with the FDA-approved chemotherapeutic enasidenib, an isocitrate dehydrogenase 2 inhibitor. They found that isocitrate dehydrogenase 2 genetic ablation or inhibition with enasidenib diverted glucose utilization away from glycolysis toward the pentose phosphate pathway, which improved activity under nutrient-starved conditions. Furthermore, central memory formation was enriched because of the shuttling of citrate into the cytosol for acetyl-CoA conversion, which altered the epigenetic landscape of CAR T cells, similar to that seen by decitabine pretreatment (Wang et al, 2021). Ultimately, metabolically reprogrammed (via enasidenib pretreatment) CD19.41BB and GD2.CD28 CAR T cells had superior antitumor activity against acute lymphoblastic leukemia and osteosarcoma preclinical models, respectively. Of importance, antitumor efficacy was further improved by daily oral administration of enasidenib (Si et al, 2024). This showcases that prolonged CAR T cell support may be necessary beyond the manufacturing process for a chemotherapeutic combination strategy to be optimal.

#### 2 Chemotherapies administered with or post chimeric antigen receptor T cell infusion

In 2016, Ruella et al (2016) published a study evaluating CD19.41BB CAR T cells with combinatorial ibrutinib against mantle cell lymphoma. Animals received CAR T cell therapy on day 7 post-tumor engraftment and daily administration of ibrutinib for the duration of the study. Combinatorial treatment resulted in long-term remission that neither monotherapy could achieve. Ibrutinib is known to inhibit T helper 2 polarization (less cytotoxic) of T cells and favor T helper 1 polarization (more cytotoxic), favoring effector function (Mhibik et al, 2019). This study concurrently showed the downregulation of T cell exhaustion markers PD-1 and CTLA4, overall improving the fitness of CAR T cells with combinatorial therapy (Ruella et al, 2016). Of importance, ibrutinib has also been shown to have a positive impact on CAR T cells in the clinic. Fraietta et al (2016) published a clinical study examining the functionality of CD19.41BB CAR T cells that were generated from chronic lymphocytic leukemia (CLL) patients who were treated with Bruton's tyrosine kinase inhibitor ibrutinib. CLL is a B cell malignancy that is susceptible to CAR T cell therapy. However, T cells from patients with CLL poorly expand ex vivo and complicate the CAR T cell manufacturing process. In the 2016 report, the authors found that CAR T cells generated from patients with prolonged ibrutinib treatment experienced significantly improved CAR T cell expansion (Fraietta et al, 2016). Improvements in CAR T cell expansion with prolonged ibrutinib treatment correlated with improved patient response to CAR T cell therapy and overall survival (Fraietta et al, 2016). The positive impact ibrutinib had on patient T cells in the clinic emphasizes the immense potential for use in combinatorial treatment strategies to improve fitness.

In another example, combinatorial treatment of CD19 and CD123 CAR T cells with the preclinical bromodomain and extraterminal motif inhibitor JQ1 improved CAR T cell fitness via reduction of CAR T cell exhaustive markers, ultimately increasing AML tumor control in mouse models (Zhong et al, 2022). The mechanism was twofold: JQ1 treatment prevented or reversed the exhaustive phenotype of CAR T cells marked by PD-1 and TIM-3 expression and diminished the level of PD-L1 expression on AML blasts. The PD-1/PD-L1 inhibitory axis relies on PD-1 on T cells recognizing PD-L1 on tumor cells. Depletion of both sides of the axis reduces the inhibitory signals received by CAR T cells and improves activation and fitness. This study highlights the ability of a chemotherapeutic to have both an activating impact on CAR T cells and simultaneous inhibitory effects on cancer cells that ultimately improve CAR T cell fitness.

### F Multiple action chemotherapies

So far, we have highlighted chemotherapy combination strategies that are primarily single-acting and modulate only one aspect of either CAR T or tumor biology but also some that have multiple mechanisms of action. For example, in a prostate cancer model using Le^Y.^CD28 CAR T cells, carboplatin induced both ECM remodeling of the tumor and promoted antitumorigenic macrophage polarization, conferring survival benefits for combination-treated mice (Porter et al, 2023). We view these multiaction combinations to be highly valuable, especially if they provide a positive benefit to CAR T cells while simultaneously negatively impacting tumor growth and survival.

A commonly used chemotherapeutic for CAR T cell combination therapy both preclinically and clinically is lenalidomide (Thieblemont et al, 2020; Wang et al, 2020; Zarei et al, 2023; Kann et al, 2024). Lenalidomide has been tested against multiple hematological and some solid malignancies in combination with B cell maturation antigen, CD19, CD20, CD23, CD133, CS1, HER2, NKG2D, and WT-1 targeting CAR T cells (Table 1). Lenalidomide is standard for multiple myeloma therapy, allowing for direct antitumor activity (Holstein et al, 2018). In combinatorial treatment with CAR T cells, lenalidomide was found to improve the expansion of CAR T cells in vivo (Kann et al, 2024)*,* improve CAR T cell effector functions (Zarei et al, 2023), and prevent early onset of CAR T cell exhaustion (Works et al, 2019). Lenalidomide has also been cited to increase IFN-*γ* and IL-2 production, reduce angiogenesis (Jin et al, 2023), improve the CAR T cell/tumor cell interaction (Tettamanti et al, 2022), and improve CAR T cell tumor infiltration (Zhang et al, 2021). It is clear that lenalidomide enhances CAR T cell therapy via direct tumor and CAR T cell mechanisms and is promisingly being actively investigated in the clinic.

Another multiaction combinatorial strategy was recently published by Gao et al (2023) targeting sphingosine 1-phosphate receptor 3 (SIPR3) on tumor cells in both breast and colon murine cancer models. High expression of SIPR3 has been heavily associated with poor patient prognosis as well as failure of checkpoint blockade therapy in cancer patients. Murine EpCAM.CD28.41BB cells were combined with TY-52156 or CAY10444 SIPR3 inhibitors in coculture with tumors and showed increased activation upon exposure to antigen (as determined using IFN and granzyme B secretion), increased memory phenotype (as determined using CD44+CD62L+ double expression), and reduced exhaustion (as determined using PD-1, TIM-3, and lymphocyte activation gene 3). The combination was more efficacious in controlling tumors in vivo than either monotherapy alone. This was further attributed to the ability of SIPR3 inhibitors to reprogram the tumor immune microenvironment by polarizing macrophages toward the M1 proinflammatory phenotype (Gao et al, 2023). M1 macrophages are not suppressive for CAR T cells and enable them to continue to effectively clear tumor cells (Rodriguez-Garcia et al, 2021). SIPR3 inhibitors are a prime example of chemotherapeutics with multiple mechanisms of action that synergize to improve tumor clearance. Additionally, it is a strategy that could likely be applied to any cancer with high SIPR3 expression.

These multiple-action drugs, in our opinion, should be prioritized as cancer researchers appreciate and try to combat the immense complexities of cancer. Targeting different cancer-associated phenotypes with a single compound may minimize disease burden and treatment-related cytotoxicity and support an environment that allows for the highest cytotoxic potential of CAR T cells.

## IV Chimeric antigen receptor T cells that address chemotherapeutic limitations

The tumor-targeting capability of CAR T cells presents a unique opportunity to further overcome some limitations of chemotherapeutics. Engineering CAR T cells to behave like nanoparticles would allow for the local administration of a secondary therapeutic alongside CAR T cell cytotoxicity. To date, this has been most explored in the context of CAR T cells that locally deliver monoclonal checkpoint blockade therapies, which are antibodies that block inhibitory signaling axes between tumor cells and CAR T cells (eg, PD-1/PD-L1, CTLA4/CD80) (Rafiq et al, 2018). CAR T cells have also been designed to secrete enzymes to modulate the tumor microenvironment. In one study, GD2.CD28.OX40 CAR T cells that secrete heparinase induced ECM degradation and improved infiltration of CAR T cells into solid tumors (Caruana et al, 2015).

Gardner et al (2022) moved this concept into the field of chemotherapeutics and developed synthetic enzyme-armed killer (“SEAKER”) CAR T cells in 2021 that secrete enzymes capable of cleaving inactive prodrugs into their active counterparts, inducing combinatorial therapy only at the site of CAR T cell localization. Specifically, SEAKER cells deploy enzymes derived from bacteria (carboxypeptidase G2 and *β*-lactamase) that would be active only on the prodrug containing the enzymatic recognition site and be innocuous to healthy tissues. The secretable enzyme sequence was cotransduced with a CD19.41BB CAR. SEAKER cells effectively activated the chemotherapeutics 5′-*O*-sulfamoyladenosine, the nitrogen mustard ZD2767, and 7-*O*-aminopropyl-7-*O*-des(morpholinopropyl)gefitinib when masked with either glutamate (cleaved by carboxypeptidase G2) or cephalosporin (cleaved by *β*-lactamase). Combinatorial treatment with SEAKER cells and respective prodrugs reduced tumor burden and extended survival against hematological and solid malignancies. Notably, SEAKER cells were able to eliminate antigen-negative cancer cells in vitro in the presence of an appropriate prodrug, whereas CAR T cells alone could not because of the low-level secretion of enzymes that are not antigen-dependent. This strategy therefore also addresses some antigen dilemma/heterogeneity challenges when local activation of potent chemotherapeutics can eliminate any tumor cells that CAR T cells cannot target because of low or absent antigen expression.

The modularity of SEAKER technology is exciting for the development of the next waves of combinatorial treatment options for CAR T cells and chemotherapeutics. Prodrugs are desirable for their ability to be specifically activated at the tumor site, limiting off-tumor toxicities (Rautio et al, 2008; Markovic et al, 2020). Prodrugs can be designed with hypoxia or acid-sensitive caps, as well as enzyme-cleavable moieties (Markovic et al, 2020). The appeal of the SEAKER cell technology is the incorporation of bacterial-derived enzyme cleavage linkers that cannot be cleaved unless SEAKER cells are present. This provides the possibility of conjugating cytotoxic drugs with tumor-targeting moieties or blood-brain penetrant moieties (Xia et al, 2021b) that can shuttle drugs to the tumor, where they will be activated by SEAKER cells. This may drastically expand the number of drugs that could be considered for brain cancer therapy that are currently limited by low blood-brain barrier penetrance.

## V Identifying chemotherapy and chimeric antigen receptor T combinations with high-throughput screening

As exemplified throughout this review, combining CAR T cell therapy with chemotherapies can be highly beneficial. Many investigators have prioritized compounds based on their known mechanisms of action that either impinge on tumor cell maintenance or positively regulate CAR T cell effector functionality. However, identifying candidates can be laborious and time-consuming, delaying potential clinical translation. High-throughput drug screening is an essential technique that can be optimized to streamline the drug identification process. Some successful preclinical CAR T cell and drug combinations reviewed herein have been identified using screening technology. Screens spanned small-molecule inhibitors, epigenetic modulators, proapoptotic molecules, kinase inhibitors, and mitochondrial targeting compounds (Dufva et al, 2020; de Billy et al, 2021; Lee et al, 2022; Zhang et al, 2023; Si et al, 2024). These drug screens identified compounds that could either modulate the immune landscape, support CAR T cell effector function and fitness, increase tumor debulking, increase targeted antigen expression, or promote a more permissive environment for CAR T cells. Collectively, these screens took a broad-scale approach to enhancing CAR T cell therapy that more rapidly identified promising candidates to be used in downstream validation.

For example, one study performed by de Billy et al (2021) identified insulin-like growth factor receptors/insulin receptors as a targetable vulnerability for diffuse midline gliomas (DMGs) through a selective kinase drug screen. This screening approach was performed by pretreating DMG cells with a singular concentration (1 μM) of 42 kinase inhibitors for an hour prior to the addition of either GD2.CD28.41BB or nontransduced CAR T cells. The tumor and T cells were cocultured in the presence or absence of the drug for 24 hours, at which point relative viability was determined using live cell imaging and metabolism-based assays. From the drug screen, linsitinib and BMS-754807 were identified as compounds that could inhibit tumor cell viability while sparing CAR T cells individually. Further validation confirmed a therapeutic benefit in the combination of linsitinib and GD2.CD28.41BB CAR T cells contributed to greater antitumor activity in both in vitro and in vivo DMG models (de Billy et al, 2021).

A more recent example of a successful compound screen was performed by Zhang et al (2023), which used a small-molecule compound library that identified JK-184, a hedgehog signaling pathway inhibitor, to complement B7-H3.CD28 CAR T cells against breast cancer cell lines. Uniquely, this study used 3 distinct approaches to conduct the screen, in which (1) tumor cells were pretreated with candidate compounds for 48 hours and then CAR T cells were added, (2) tumor cells were incubated with compounds for 48 hours in the absence of CAR T cells, or (3) CAR T cells were incubated with the candidate compounds for 24 hours in the absence of tumor cells. This multipronged approach allowed for specific characterization of drug-induced effects on each cell type individually while also being able to determine a combinatorial benefit. Following identification, tumor cells and CAR T cells treated with candidate compounds were subjected to RNA sequencing and gene pathway enrichment analysis to identify differentially expressed genes that may be mechanistically contributing to the observed benefit. Later experiments confirmed that JK184 enhanced B7-H3.CD28 infiltration and reshaped the tumor milieu by inhibiting immunosuppressive myeloid populations (Zhang et al, 2023).

A main consideration for performing a drug screen is the vast heterogeneity of cancer types that drive the deficiencies of CAR T cell therapy. For instance, many CAR T cell therapies targeting blood cancers fail to prevent relapse because of antigen downregulation or loss on the surface of cancer cells (Mishra et al, 2024). This is not often a challenge with solid and brain cancers, but poor infiltration and quick progression of exhaustion are (Marofi et al, 2021). Knowing the primary challenges CAR T cells will face against a given tumor type is crucial. Then, targeted drug screens with appropriate readouts should be performed to identify candidates for combinatorial therapy. Ideally, because more screens are performed, drug candidates will surface that are effective in combination with CAR T cells regardless of CAR construction against multiple cancers. However, because the field enters further into investigations of this nature, it is imperative to perform unique screens for each tumor type and different CAR targets and structures. Screens should additionally be designed in a way that the impact of a chemotherapeutic on both the tumor and CAR T cells can be assessed.

## VI Critical considerations for chemotherapy and chimeric antigen receptor T cell combination therapy

Herein, we have shown the vast number of mechanisms by which combinatorial chemotherapy can overcome challenges faced by CAR T cell therapy. Chemotherapeutics can increase surface levels of targetable surface antigens; assist cytotoxic CAR T cells via tumor debulking; improve CAR T cell homing and penetrance into solid tumor masses; rewire CAR T cell transcription, epigenetic, and metabolic programs to improve fitness and persistence; and combat other cells in the tumor immune microenvironment that are hostile toward CAR T cells. In some cases, a single chemotherapeutic agent will address multiple CAR T cell deficiencies with the ability to target programs in tumor cells or tumor-associated immune cells that are deleterious and in the CAR T cells that are beneficial. Additionally, here are many instances of CAR T cell and chemotherapy combination strategies in the clinic (Al-Haideri et al, 2022). As more combination strategies are being investigated, there are many different aspects that must be considered and addressed in the preclinical stage for successful implementation in the clinic.

### A Thorough preclinical efficacy and safety testing

Thorough preclinical evaluation of combinatorial CAR T and chemotherapy combination strategies is imperative for successful translation into the clinic. For many CAR T cell preclinical studies, human tumors are orthotopically implanted into immunocompromised mice prior to treatment with human CAR T cells. Immunodeficiency of mice ensures that human tumors and CAR T cells will not be rejected by the murine immune system. While this methodology confirms that human CAR T cells successfully target human tumors in a living system, true safety testing is not possible in this setting. Some CAR targets highly expressed on tumor cells can be expressed on healthy tissues (eg, GD2 and Epha2), which may pose on-target off-tumor health risks (Zhao et al, 2021; Machy et al, 2023). Additionally, immunocompromised mice lack the full tumor immune microenvironment that may significantly change the outcome of CAR T cell therapy (Haydar et al, 2023). Similarly, chemotherapeutics must undergo rigorous safety testing to ensure that the benefit of the therapy outweighs the possible risks. Even if both therapies are proved to be safe separately, safety testing of combination therapy must still be performed because of possible alterations of safety profiles of either the chemotherapeutic agents or CAR T cells. It is imperative to perform preclinical safety and efficacy studies of combinatorial strategies in syngeneic models with murine tumors that mimic human pathology and CAR T cells derived from murine T lymphocytes so that the safety of both chemotherapeutic agents and CAR T cells can be evaluated in the presence of the endogenous immune system.

### B Optimizing delivery approach

**Fig. 3:**
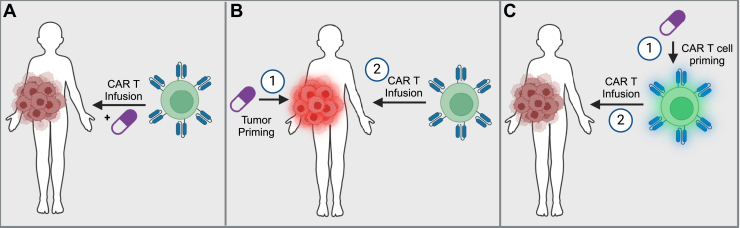
Treatment options for CAR T and chemotherapy combination strategies. (A) CAR T cells and chemotherapeutic agents are administered simultaneously; chemotherapeutic agents may be continuously administered postinfusion. (B) Tumor cells are (1) primed prior to (2) CAR T cell administration. (C) CAR T cells are (1) primed prior to (2) infusion. Other timelines may be considered that are a combination of these 3 basic approaches.

An additional challenge for chemotherapy and CAR T cell combinatorial approaches is identifying an appropriate dosing timeline. In the most traditional sense of a combinatory strategy, 2 different therapies can be administered simultaneously to a patient (Fig. 3A). This strategy may be ideal when considering a combinatorial strategy that will most profoundly impact CAR T cells within the tumor microenvironment or for chemotherapeutics that act on both CAR T cells and a component of the tumor. However, we have discussed in this review that there is also a benefit to either priming tumors (Fig. 3B) or CAR T cells (Fig. 3C) with chemotherapeutics prior to infusion, depending on the mechanism of the chemotherapeutic agents.

For chemotherapeutics that exclusively target the tumor, pretreatment of patients with chemotherapeutics prior to CAR T cell administration is a viable option. For example, chemotherapeutics that debulk tumors or increase surface antigen levels are likely to be most beneficial if administered sometime before CAR T cell treatment. However, it is crucial to consider the impact this will have on the health of patient T cells. A 2019 study by Das et al (2019) studied the naïve populations of T cells collected from the periphery of patients at the beginning of cancer diagnosis and throughout rounds of standard chemotherapy for multiple cancers. The naïve phenotype was singled out as a proportion of naïve cells in CAR T cell infusion products that have been shown to correlate to treatment efficacy. A total of 195 pediatric patients with cancer across 10 different diagnoses (including hematological and solid malignancies) were enrolled in the study. CAR T cells were generated from peripheral blood mononuclear cells, surface phenotyping was performed to identify the proportion of naïve cells and ex vivo expansion was used as the primary benchmark for assessing CAR T cell functionality/health. Generally, regardless of cancer type, CAR T cell health was superior at diagnosis as compared with following any amount of chemotherapy and was dependent on the initial proportion of naïve/central memory CAR T cells. These data highlight that the timing of blood collection for the generation of CAR T cells for patients who might receive CAR T cell therapy following standard chemotherapy needs to be carefully considered to optimize efficacy. Potential combinatorial strategies should also consider whether chemotherapy might have a negative impact on overall T cell health if administered first.

For chemotherapeutics that exclusively act on CAR T cells, adding the agent to the manufacturing process is readily approved, as discussed in the “Fitness” section above. However, it is possible that for some inhibitors acting on CAR T cells, like enasidenib, the maximum therapeutic benefit is achieved with pretreatment in the manufacturing process and continued administration following CAR T cell infusion (Si et al, 2024). Investigations into each possibility and combinations thereof are necessary to optimize the strategy.

### C Optimizing dosing amounts

Another consideration for appropriate dosing strategy is the overall amount of both chemotherapeutic agent and CAR T cell administration that is optimal for efficacy. If truly synergistic, it is hopeful that the doses of both agents can be minimized to avoid off-tumor toxicities. It is well established that chemotherapies have intense systemic toxicities that lead to sickness while on chemotherapeutic regimens as well as long-lasting effects, including an increased risk of developing other cancers (Brower, 2013; Demoor-Goldschmidt and de Vathaire, 2019). When CAR T cells are activated by antigen recognition, they secrete cytokines that elicit inflammatory responses. When tumors are large and CAR T cells are working effectively, this can cause patients to experience symptoms of sickness that fall on a grading scale of cytokine release syndrome and immune effector cell-associated neurotoxicity syndrome (Morris et al, 2022). Most symptoms are readily managed through treatment with tocilizumab and/or steroids, and the chemical drug ruxolitinib has also been shown to mitigate cytokine release syndromes when refractory to steroids (Pan et al, 2021). Optimal synergistic combinatorial therapies of chemotherapeutics and CAR T cells may reduce the dose required of both agents, resulting in maximum efficacy with minimal side effects.

Another crucial consideration for combinatorial strategies is whether a given chemotherapeutic agent will impair the ability of CAR T cells to successfully engraft and proliferate in response to antigens. A prominent consideration for this is when combining CAR T cells with standard-of-care therapy. Dexamethasone is commonly used in GBM treatment schema. As such, it makes sense to combine dexamethasone and CAR T cell therapies. However, dexamethasone has been shown to critically reduce CAR T cell efficacy at high doses but remain relatively inert at lower doses (Brummer et al, 2022). This highlights the importance of optimizing both timing and dosing strategies when combining CAR T cells and chemotherapeutics.

## VII Summary and conclusions

Overall, combinatorial CAR T cell and chemotherapeutic treatment strategies are promising for overcoming both CAR T cell and chemotherapeutic deficiencies. These possibilities are largely unexplored for many cancer types and CAR designs, representing an open field for further investigation. While there are many aspects to optimize, such as CAR structure, chemotherapeutic target, and dosing strategy, combinatorial therapies are showing early preclinical success for overcoming limitations in CAR T cell therapies to ultimately improve survival outcomes for patients. Finally, implementing a combinatorial treatment approach in the clinic has the potential to be faster and cheaper, especially if both therapeutics have been FDA-approved.

## Conflict of interest

No author has an actual or perceived conflict of interest with the contents of this article.

## References

[bib1] Abate-Daga D., Davila M.L. (2016). CAR models: next-generation CAR modifications for enhanced T-cell function. Mol Ther Oncolytics.

[bib2] Abbott R.C., Cross R.S., Jenkins M.R. (2020). Finding the keys to the CAR: identifying novel target antigens for T cell redirection immunotherapies. Int J Mol Sci.

[bib3] Aboelella N.S., Brandle C., Okoko O., Gazi M.Y., Ding Z.C., Xu H., Gorman G., Bollag R., Davila M.L., Bryan L.J. (2022). Indomethacin-induced oxidative stress enhances death receptor 5 signaling and sensitizes tumor cells to adoptive T-cell therapy. J Immunother Cancer.

[bib4] Al-Haideri M., Tondok S.B., Safa S.H., Maleki A.H., Rostami S., Jalil A.T., Al-Gazally M.E., Alsaikhan F., Rizaev J.A., Mohammad T.A.M. (2022). CAR-T cell combination therapy: the next revolution in cancer treatment. Cancer Cell Int.

[bib5] Albinger N., Hartmann J., Ullrich E. (2021). Current status and perspective of CAR-T and CAR-NK cell therapy trials in Germany. Gene Ther.

[bib6] Amini L., Silbert S.K., Maude S.L., Nastoupil L.J., Ramos C.A., Brentjens R.J., Sauter C.S., Shah N.N., Abou-el-Enein M. (2022). Preparing for CAR T cell therapy: patient selection, bridging therapies and lymphodepletion. Nat Rev Clin Oncol.

[bib7] Anand U., Dey A., Chandel A.K.S., Sanyal R., Mishra A., Pandey D.K., De Falco V., Upadhyay A., Kandimalla R., Chaudhary A. (2023). Cancer chemotherapy and beyond: current status, drug candidates, associated risks and progress in targeted therapeutics. Genes Dis.

[bib8] Asmamaw Dejenie T., Tiruneh G.M.M., Dessie Terefe G., Tadele Admasu F., Wale Tesega W., Chekol Abebe E. (2022). Current updates on generations, approvals, and clinical trials of CAR T-cell therapy. Hum Vaccin Immunother.

[bib9] Bell M., Lange S., Sejdiu B.I., Ibanez J., Shi H., Sun X., Meng X., Nguyen P., Sutton M., Wagner J. (2023). Modular chimeric cytokine receptors with leucine zippers enhance the antitumour activity of CAR T cells via JAK/STAT signalling. Nat Biomed Eng.

[bib10] Berahovich R., Liu X., Zhou H., Tsadik E., Xu S., Golubovskaya V., Wu L. (2019). Hypoxia selectively impairs CAR-T cells in vitro. Cancers (Basel).

[bib11] Bergaggio E., Tai W.-T., Aroldi A., Mecca C., Landoni E., Nüesch M., Mota I., Metovic J., Molinaro L., Ma L. (2023). ALK inhibitors increase ALK expression and sensitize neuroblastoma cells to ALK.CAR-T cells. Cancer Cell.

[bib12] Bied M., Ho W.W., Ginhoux F., Blériot C. (2023). Roles of macrophages in tumor development: a spatiotemporal perspective. Cell Mol Immunol.

[bib13] Brassard D.L., Grace M.J., Bordens R.W. (2002). Interferon-alpha as an immunotherapeutic protein. J Leukoc Biol.

[bib14] Breman E., Demoulin B., Agaugué S., Mauën S., Michaux A., Springuel L., Houssa J., Huberty F., Jacques-Hespel C., Marchand C. (2018). Overcoming target driven fratricide for T cell therapy. Front Immunol.

[bib15] Brower V. (2013). Tracking chemotherapy’s effects on secondary cancers. J Natl Cancer Inst.

[bib16] Brummel K., Eerkens A.L., de Bruyn M., Nijman H.W. (2023). Tumour-infiltrating lymphocytes: from prognosis to treatment selection. Br J Cancer.

[bib17] Brummer A.B., Yang X., Ma E., Gutova M., Brown C.E., Rockne R.C. (2022). Dose-dependent thresholds of dexamethasone destabilize CAR T-cell treatment efficacy. PLoS Comput Biol.

[bib18] Cappell K.M., Kochenderfer J.N. (2021). A comparison of chimeric antigen receptors containing CD28 versus 4-1BB costimulatory domains. Nat Rev Clin Oncol.

[bib19] Carpenter E.L., Mossé Y.P. (2012). Targeting ALK in neuroblastoma—preclinical and clinical advancements. Nat Rev Clin Oncol.

[bib20] Caruana I., Savoldo B., Hoyos V., Weber G., Liu H., Kim E.S., Ittmann M.M., Marchetti D., Dotti G. (2015). Heparanase promotes tumor infiltration and antitumor activity of CAR-redirected T lymphocytes. Nat Med.

[bib21] Castellarin M., Sands C., Da T., Scholler J., Graham K., Buza E., Fraietta J.A., Zhao Y., June C.H. (2020). A rational mouse model to detect on-target, off-tumor CAR T cell toxicity. JCI Insight.

[bib22] Chakraborty S., Rahman T. (2012). The difficulties in cancer treatment. Ecancermedicalscience.

[bib23] Chmielewski M., Abken H. (2015). TRUCKs: the fourth generation of CARs. Expert Opin Biol Ther.

[bib24] Chow A., Perica K., Klebanoff C.A., Wolchok J.D. (2022). Clinical implications of T cell exhaustion for cancer immunotherapy. Nat Rev Clin Oncol.

[bib25] Das R.K., Vernau L., Grupp S.A., Barrett D.M. (2019). Naïve T-cell deficits at diagnosis and after chemotherapy impair cell therapy potential in pediatric cancers. Cancer Discov.

[bib26] de Billy E., Pellegrino M., Orlando D., Pericoli G., Ferretti R., Businaro P., Ajmone-Cat M.A., Rossi S., Petrilli L.L., Maestro N. (2021). Dual IGF1R/IR inhibitors in combination with GD2-CAR T-cells display a potent anti-tumor activity in diffuse midline glioma H3K27M-mutant. Neuro Oncol.

[bib27] Demoor-Goldschmidt C., de Vathaire F. (2019). Review of risk factors of secondary cancers among cancer survivors. Br J Radiol.

[bib28] Doan A.E., Mueller K.P., Chen A.Y., Rouin G.T., Chen Y., Daniel B., Lattin J., Markovska M., Mozarsky B., Arias-Umana J. (2024). FOXO1 is a master regulator of memory programming in CAR T cells. Nature.

[bib29] Dufva O., Koski J., Maliniemi P., Ianevski A., Klievink J., Leitner J., Pölönen P., Hohtari H., Saeed K., Hannunen T. (2020). Integrated drug profiling and CRISPR screening identify essential pathways for CAR T-cell cytotoxicity. Blood.

[bib30] El Khawanky N., Hughes A., Yu W., Myburgh R., Matschulla T., Taromi S., Aumann K., Clarson J., Vinnakota J.M., Shoumariyeh K. (2021). Demethylating therapy increases anti-CD123 CAR T cell cytotoxicity against acute myeloid leukemia. Nat Commun.

[bib31] Eno J. (2017). Immunotherapy through the years. J Adv Pract Oncol.

[bib32] Eshhar Z., Waks T., Gross G., Schindler D.G. (1993). Specific activation and targeting of cytotoxic lymphocytes through chimeric single chains consisting of antibody-binding domains and the gamma or zeta subunits of the immunoglobulin and T-cell receptors. Proce Natl Acad Sci U SA.

[bib33] Fabrizio V.A., Boelens J.J., Mauguen A., Baggott C., Prabhu S., Egeler E., Mavroukakis S., Pacenta H., Phillips C.L., Rossoff J. (2022). Optimal fludarabine lymphodepletion is associated with improved outcomes after CAR T-cell therapy. Blood Adv.

[bib34] Farrukh S., Habib S., Rafaqat A., Sarfraz Z., Sarfraz A., Sarfraz M., Robles-Velasco K., Felix M., Cherrez-Ojeda I. (2023). Emerging therapeutic strategies for diffuse intrinsic pontine glioma: a systematic review. Healthcare (Basel).

[bib35] Feins S., Kong W., Williams E.F., Milone M.C., Fraietta J.A. (2019). An introduction to chimeric antigen receptor (CAR) T-cell immunotherapy for human cancer. Am J Hematol.

[bib36] Finney H.M., Akbar A.N., Lawson A.D. (2004). Activation of resting human primary T cells with chimeric receptors: costimulation from CD28, inducible costimulator, CD134, and CD137 in series with signals from the TCR zeta chain. J Immunol.

[bib37] Finney H.M., Lawson A.D., Bebbington C.R., Weir A.N. (1998). Chimeric receptors providing both primary and costimulatory signaling in T cells from a single gene product. J Immunol.

[bib38] Flugel C.L., Majzner R.G., Krenciute G., Dotti G., Riddell S.R., Wagner D.L., Abou-El-Enein M. (2023). Overcoming on-target, off-tumour toxicity of CAR T cell therapy for solid tumours. Nat Rev Clin Oncol.

[bib39] Fraietta J.A., Beckwith K.A., Patel P.R., Ruella M., Zheng Z., Barrett D.M., Lacey S.F., Melenhorst J.J., McGettigan S.E., Cook D.R. (2016). Ibrutinib enhances chimeric antigen receptor T-cell engraftment and efficacy in leukemia. Blood.

[bib40] Fu Q., Zheng Y., Fang W., Zhao Q., Zhao P., Liu L., Zhai Y., Tong Z., Zhang H., Lin M. (2023). RUNX-3-expressing CAR T cells targeting glypican-3 in patients with heavily pretreated advanced hepatocellular carcinoma: a phase I trial. eClinicalMedicine.

[bib41] Fujiwara K., Tsunei A., Kusabuka H., Ogaki E., Tachibana M., Okada N. (2020). Hinge and transmembrane domains of chimeric antigen receptor regulate receptor expression and signaling threshold. Cells.

[bib42] Gao G., Liao W., Shu P., Ma Q., He X., Zhang B., Qin D., Wang Y. (2023). Targeting sphingosine 1-phosphate receptor 3 inhibits T-cell exhaustion and regulates recruitment of proinflammatory macrophages to improve antitumor efficacy of CAR-T cells against solid tumor. J Immunother Cancer.

[bib43] Gardner T.J., Lee J.P., Bourne C.M., Wijewarnasuriya D., Kinarivala N., Kurtz K.G., Corless B.C., Dacek M.M., Chang A.Y., Mo G. (2022). Engineering CAR-T cells to activate small-molecule drugs in situ. Nat Chem Biol.

[bib44] Goldsmith K.C., Park J.R., Kayser K., Malvar J., Chi Y.Y., Groshen S.G., Villablanca J.G., Krytska K., Lai L.M., Acharya P.T. (2023). Lorlatinib with or without chemotherapy in ALK-driven refractory/relapsed neuroblastoma: phase 1 trial results. Nature Med.

[bib45] Grupp S.A., Kalos M., Barrett D., Aplenc R., Porter D.L., Rheingold S.R., Teachey D.T., Chew A., Hauck B., Wright J.F. (2013). Chimeric antigen receptor-modified T cells for acute lymphoid leukemia. N Engl J Med.

[bib46] Guest R.D., Hawkins R.E., Kirillova N., Cheadle E.J., Arnold J., O'Neill A., Irlam J., Chester K.A., Kemshead J.T., Shaw D.M. (2005). The role of extracellular spacer regions in the optimal design of chimeric immune receptors: evaluation of four different scFvs and antigens. J Immunother.

[bib47] Han Y., Sun B., Cai H., Xuan Y. (2021). Simultaneously target of normal and stem cells-like gastric cancer cells via cisplatin and anti-CD133 CAR-T combination therapy. Cancer Immunol Immunother.

[bib48] Hargrave A., Mustafa A.S., Hanif A., Tunio J.H., Hanif S.N.M. (2023). Recent Advances in Cancer Immunotherapy with a Focus on FDA-Approved Vaccines and Neoantigen-Based Vaccines. Vaccines (Basel).

[bib49] Harrer D.C., Schenkel C., Berking C., Herr W., Abken H., Dörrie J., Schaft N. (2022). Decitabine-mediated upregulation of CSPG4 in ovarian carcinoma cells enables targeting by CSPG4-specific CAR-T cells. Cancers (Basel).

[bib50] Hasan T., Pasala A.R., Hassan D., Hanotaux J., Allan D.S., Maganti H.B. (2024). Homing and engraftment of hematopoietic stem cells following transplantation: a pre-clinical perspective. Currt Oncol.

[bib51] Haydar D., Ibañez-Vega J., Crawford J.C., Chou C.H., Guy C.S., Meehl M., Yi Z., Perry S., Laxton J., Cunningham T. (2023). CAR T-cell design-dependent remodeling of the brain tumor immune microenvironment modulates tumor-associated macrophages and anti-glioma activity. Cancer Res Commun.

[bib52] He Z., Zhang S. (2021). Tumor-associated macrophages and their functional transformation in the hypoxic tumor microenvironment. Front Immunol.

[bib53] Hebbar N., Epperly R., Vaidya A., Thanekar U., Moore S.E., Umeda M., Ma J., Patil S.L., Langfitt D., Huang S. (2022). CAR T cells redirected to cell surface GRP78 display robust anti-acute myeloid leukemia activity and do not target hematopoietic progenitor cells. Nat Commun.

[bib54] Heczey A., Louis C.U., Savoldo B., Dakhova O., Durett A., Grilley B., Liu H., Wu M.F., Mei Z., Gee A. (2017). CAR T cells administered in combination with lymphodepletion and PD-1 inhibition to patients with neuroblastoma. Mol Ther.

[bib55] Henke E., Nandigama R., Ergün S. (2019). Extracellular matrix in the tumor microenvironment and its impact on cancer therapy. Front Mol Biosci.

[bib56] Holstein S.A., Suman V.J., McCarthy P.L. (2018). Update on the role of lenalidomide in patients with multiple myeloma. Ther Adv Hematol.

[bib57] Huang B., Pan P.Y., Li Q., Sato A.I., Levy D.E., Bromberg J., Divino C.M., Chen S.H. (2006). Gr-1+CD115+ immature myeloid suppressor cells mediate the development of tumor-induced T regulatory cells and T-cell anergy in tumor-bearing host. Cancer Res.

[bib58] Ibanez J., Hebbar N., Thanekar U., Yi Z., Houke H., Ward M., Nevitt C., Tian L., Mack S.C., Sheppard H. (2023). GRP78-CAR T cell effector function against solid and brain tumors is controlled by GRP78 expression on T cells. Cell Rep Med.

[bib59] Jain N., Zhao Z., Feucht J., Koche R., Iyer A., Dobrin A., Mansilla-Soto J., Yang J., Zhan Y., Lopez M. (2023). TET2 guards against unchecked BATF3-induced CAR T cell expansion. Nature.

[bib60] Jain N., Zhao Z., Koche R.P., Antelope C., Gozlan Y., Montalbano A., Brocks D., Lopez M., Dobrin A., Shi Y. (2024). Disruption of SUV39H1-mediated H3K9 methylation sustains CAR T-cell function. Cancer Discov.

[bib61] Jayaraman J., Mellody M.P., Hou A.J., Desai R.P., Fung A.W., Pham A.H.T., Chen Y.Y., Zhao W. (2020). CAR-T design: elements and their synergistic function. EBioMedicine.

[bib62] Jetani H., Garcia-Cadenas I., Nerreter T., Thomas S., Rydzek J., Meijide J.B., Bonig H., Herr W., Sierra J., Einsele H. (2018). CAR T-cells targeting FLT3 have potent activity against FLT3−ITD+ AML and act synergistically with the FLT3-inhibitor crenolanib. Leukemia.

[bib63] Jin Z., Xiang R., Qing K., Li D., Liu Z., Li X., Zhu H., Zhang Y., Wang L., Xue K. (2023). Lenalidomide overcomes the resistance to third-generation CD19-CAR-T cell therapy in preclinical models of diffuse large B-cell lymphoma. Cell Oncol (Dordr).

[bib64] Johnson A., Townsend M., O'Neill K. (2022). Tumor microenvironment immunosuppression: a roadblock to CAR T-cell advancement in solid tumors. Cells.

[bib65] Kann M.C., Schneider E.M., Almazan A.J., Lane I.C., Bouffard A.A., Supper V.M., Takei H.N., Tepper A., Leick M.B., Larson R.C. (2024). Chemical genetic control of cytokine signaling in CAR-T cells using lenalidomide-controlled membrane-bound degradable IL-7. Leukemia.

[bib66] Kochenderfer J.N., Dudley M.E., Kassim S.H., Somerville R.P., Carpenter R.O., Stetler-Stevenson M., Yang J.C., Phan G.Q., Hughes M.S., Sherry R.M. (2015). Chemotherapy-refractory diffuse large B-cell lymphoma and indolent B-cell malignancies can be effectively treated with autologous T cells expressing an anti-CD19 chimeric antigen receptor. J Clin Oncol.

[bib67] Korman A.J., Garrett-Thomson S.C., Lonberg N. (2022). The foundations of immune checkpoint blockade and the ipilimumab approval decennial. Nat Rev Drug Discov.

[bib68] Krenciute G., Prinzing B.L., Yi Z., Wu M.F., Liu H., Dotti G., Balyasnikova I.V., Gottschalk S. (2017). Transgenic expression of IL15 improves antiglioma activity of IL13Rα2-CAR T cells but results in antigen loss variants. Cancer Immunol Res.

[bib69] Lanitis E., Irving M., Coukos G. (2015). Targeting the tumor vasculature to enhance T cell activity. Curr Opin Immunol.

[bib70] Laskowski T.J., Biederstädt A., Rezvani K. (2022). Natural killer cells in antitumour adoptive cell immunotherapy. Nat Rev Cancer.

[bib71] Lee Y.G., Guruprasad P., Ghilardi G., Pajarillo R., Sauter C.T., Patel R., Ballard H.J., Hong S.J., Chun I., Yang N. (2022). Modulation of BCL-2 in both T cells and tumor cells to enhance chimeric antigen receptor T-cell immunotherapy against cancer. Cancer Discov.

[bib72] Leick M.B., Silva H., Scarfò I., Larson R., Choi B.D., Bouffard A.A., Gallagher K., Schmidts A., Bailey S.R., Kann M.C. (2022). Non-cleavable hinge enhances avidity and expansion of CAR-T cells for acute myeloid leukemia. Cancer Cell.

[bib73] Li H., Ding J., Lu M., Liu H., Miao Y., Li L., Wang G., Zheng J., Pei D., Zhang Q. (2020). CAIX-specific CAR-T Cells and sunitinib show synergistic effects against metastatic renal cancer models. J Immunother.

[bib74] Lickefett B., Chu L., Ortiz-Maldonado V., Warmuth L., Barba P., Doglio M., Henderson D., Hudecek M., Kremer A., Markman J. (2023). Lymphodepletion - an essential but undervalued part of the chimeric antigen receptor T-cell therapy cycle. Front Immunol.

[bib75] López-Cobo S., Fuentealba J.R., Gueguen P., Bonté P.-E., Tsalkitzi K., Chacón I., Glauzy S., Bohineust A., Biquand A., Silva L. (2024). SUV39H1 Ablation Enhances long-term CAR T function in solid tumors. Cancer Discov.

[bib76] Lorscheider M., Gaudin A., Nakhlé J., Veiman K.-L., Richard J., Chassaing C. (2021). Challenges and opportunities in the delivery of cancer therapeutics: update on recent progress. Ther Deliv.

[bib164] Lu R., Zhao G., Yang Y., Jiang Z., Cai J., Hu H. (2019). Inhibition of CD133 overcomes cisplatin resistance through inhibiting PI3K/AKT/mTOR signaling pathway and autophagy in CD133-positive gastric cancer cells. Technol Cancer Res Treat.

[bib77] Lynn R.C., Weber E.W., Sotillo E., Gennert D., Xu P., Good Z., Anbunathan H., Lattin J., Jones R., Tieu V. (2019). c-Jun overexpression in CAR T cells induces exhaustion resistance. Nature.

[bib78] Machy P., Mortier E., Birklé S. (2023). Biology of GD2 ganglioside: implications for cancer immunotherapy. Front Pharmacol.

[bib79] Makuku R., Khalili N., Razi S., Keshavarz-Fathi M., Rezaei N. (2021). Current and future perspectives of PD-1/PDL-1 blockade in cancer immunotherapy. J Immunol Res.

[bib80] Markovic M., Ben-Shabat S., Dahan A. (2020). Prodrugs for improved drug delivery: lessons learned from recently developed and marketed products. Pharmaceutics.

[bib81] Markowitz J., Wang J., Vangundy Z., You J., Yildiz V., Yu L., Foote I.P., Branson O.E., Stiff A.R., Brooks T.R. (2017). Nitric oxide mediated inhibition of antigen presentation from DCs to CD4(+) T cells in cancer and measurement of STAT1 nitration. Sci Rep.

[bib82] Marofi F., Motavalli R., Safonov V.A., Thangavelu L., Yumashev A.V., Alexander M., Shomali N., Chartrand M.S., Pathak Y., Jarahian M. (2021). CAR T cells in solid tumors: challenges and opportunities. Stem Cell Res Ther.

[bib83] Mestermann K., Giavridis T., Weber J., Rydzek J., Frenz S., Nerreter T., Mades A., Sadelain M., Einsele H., Hudecek M. (2019). The tyrosine kinase inhibitor dasatinib acts as a pharmacologic on/off switch for CAR T cells. Sci Transl Med.

[bib84] Mhibik M., Wiestner A., Sun C. (2019). Harnessing the effects of BTKi on T cells for effective immunotherapy against CLL. Int J Mol Sci.

[bib85] Mishra A., Maiti R., Mohan P., Gupta P. (2024). Antigen loss following CAR-T cell therapy: Mechanisms, implications, and potential solutions. Eur J Haematol.

[bib86] Morgan R.A., Yang J.C., Kitano M., Dudley M.E., Laurencot C.M., Rosenberg S.A. (2010). Case report of a serious adverse event following the administration of T cells transduced with a chimeric antigen receptor recognizing ERBB2. Mol Ther.

[bib87] Morotti M., Albukhari A., Alsaadi A., Artibani M., Brenton J.D., Curbishley S.M., Dong T., Dustin M.L., Hu Z., McGranahan N. (2021). Promises and challenges of adoptive T-cell therapies for solid tumours. Br J Cancer.

[bib88] Morris E.C., Neelapu S.S., Giavridis T., Sadelain M. (2022). Cytokine release syndrome and associated neurotoxicity in cancer immunotherapy. Nat Rev Immunol.

[bib89] Naval J., de Miguel D., Gallego-Lleyda A., Anel A., Martinez-Lostao L. (2019). Importance of TRAIL molecular anatomy in receptor oligomerization and signaling. Implications for cancer therapy. Cancers (Basel).

[bib90] Nguyen P., Okeke E., Clay M., Haydar D., Justice J., O'Reilly C., Pruett-Miller S., Papizan J., Moore J., Zhou S. (2020). Route of 41BB/41BBL costimulation determines effector function of B7-H3-CAR.CD28ζ T cells. Mol Ther Oncolytics.

[bib91] Nurgali K., Jagoe R.T., Abalo R. (2018). Editorial: adverse effects of cancer chemotherapy: anything new to improve tolerance and reduce sequelae?. Front Pharmacol.

[bib92] Pan J., Deng B., Ling Z., Song W., Xu J., Duan J., Wang Z., Chang A.H., Feng X., Tan Y. (2021). Ruxolitinib mitigates steroid-refractory CRS during CAR T therapy. J Cell Mol Med.

[bib93] Park J.A., Espinosa-Cotton M., Guo H.F., Monette S., Cheung N.V. (2023). Targeting tumor vasculature to improve antitumor activity of T cells armed ex vivo with T cell engaging bispecific antibody. J Immunother Cancer.

[bib94] Peng J.-J., Wang L., Li Z., Ku C.-L., Ho P.-C. (2023). Metabolic challenges and interventions in CAR T cell therapy. Science Immunology.

[bib95] Porter D.L., Levine B.L., Kalos M., Bagg A., June C.H. (2011). Chimeric antigen receptor-modified T cells in chronic lymphoid leukemia. N Engl J Med.

[bib96] Porter L.H., Zhu J.J., Lister N.L., Harrison S.G., Keerthikumar S., Goode D.L., Urban R.Q., Byrne D.J., Azad A., Vela I. (2023). Low-dose carboplatin modifies the tumor microenvironment to augment CAR T cell efficacy in human prostate cancer models. Nat Commun.

[bib97] Prinzing B., Zebley C.C., Petersen C.T., Fan Y., Anido A.A., Yi Z., Nguyen P., Houke H., Bell M., Haydar D. (2021). Deleting DNMT3A in CAR T cells prevents exhaustion and enhances antitumor activity. Sci Transl Med.

[bib98] Quail D.F., Joyce J.A. (2013). Microenvironmental regulation of tumor progression and metastasis. Nat Med.

[bib99] Raber P.L., Thevenot P., Sierra R., Wyczechowska D., Halle D., Ramirez M.E., Ochoa A.C., Fletcher M., Velasco C., Wilk A. (2014). Subpopulations of myeloid-derived suppressor cells impair T cell responses through independent nitric oxide-related pathways. Int J Cancer.

[bib100] Rafiq S., Hackett C.S., Brentjens R.J. (2020). Engineering strategies to overcome the current roadblocks in CAR T cell therapy. Nat Rev Clin Oncol.

[bib101] Rafiq S., Yeku O.O., Jackson H.J., Purdon T.J., van Leeuwen D.G., Drakes D.J., Song M., Miele M.M., Li Z., Wang P. (2018). Targeted delivery of a PD-1-blocking scFv by CAR-T cells enhances anti-tumor efficacy in vivo. Nat Biotechnol.

[bib102] Ramos C.A., Rouce R., Robertson C.S., Reyna A., Narala N., Vyas G., Mehta B., Zhang H., Dakhova O., Carrum G. (2018). In Vivo fate and activity of second- versus third-generation CD19-specific CAR-T cells in B cell non-Hodgkin's lymphomas. Mol Ther.

[bib103] Rautio J., Kumpulainen H., Heimbach T., Oliyai R., Oh D., Järvinen T., Savolainen J. (2008). Prodrugs: design and clinical applications. Nat Rev Drug Discov.

[bib104] Rodriguez-Garcia A., Lynn R.C., Poussin M., Eiva M.A., Shaw L.C., O’Connor R.S., Minutolo N.G., Casado-Medrano V., Lopez G., Matsuyama T. (2021). CAR-T cell-mediated depletion of immunosuppressive tumor-associated macrophages promotes endogenous antitumor immunity and augments adoptive immunotherapy. Nat Commu.

[bib105] Ruella M., Kenderian S.S., Shestova O., Fraietta J.A., Qayyum S., Zhang Q., Maus M.V., Liu X., Nunez-Cruz S., Klichinsky M. (2016). The addition of the BTK inhibitor ibrutinib to anti-CD19 chimeric antigen receptor T cells (CART19) improves responses against mantle cell lymphoma. Clin Cancer Res.

[bib106] Rupp L.J., Schumann K., Roybal K.T., Gate R.E., Ye C.J., Lim W.A., Marson A. (2017). CRISPR/Cas9-mediated PD-1 disruption enhances anti-tumor efficacy of human chimeric antigen receptor T cells. Sci Rep.

[bib107] Sadelain M., Brentjens R., Rivière I. (2013). The basic principles of chimeric antigen receptor design. Cancer Discov.

[bib108] Salmon H., Franciszkiewicz K., Damotte D., Dieu-Nosjean M.C., Validire P., Trautmann A., Mami-Chouaib F., Donnadieu E. (2012). Matrix architecture defines the preferential localization and migration of T cells into the stroma of human lung tumors. J Clin Invest.

[bib109] Scharping N.E., Menk A.V., Moreci R.S., Whetstone R.D., Dadey R.E., Watkins S.C., Ferris R.L., Delgoffe G.M. (2016). The tumor microenvironment represses T Cell mitochondrial biogenesis to drive intratumoral T cell metabolic insufficiency and dysfunction. Immunity.

[bib110] Schirrmacher V. (2019). From chemotherapy to biological therapy: A review of novel concepts to reduce the side effects of systemic cancer treatment (Review). Int J Oncol.

[bib111] Scott E.C., Baines A.C., Gong Y., Moore R., Pamuk G.E., Saber H., Subedee A., Thompson M.D., Xiao W., Pazdur R. (2023). Trends in the approval of cancer therapies by the FDA in the twenty-first century. Nat Rev Drug Discov.

[bib112] Sethumadhavan S., Silva M., Philbrook P., Nguyen T., Hatfield S.M., Ohta A., Sitkovsky M.V. (2017). Hypoxia and hypoxia-inducible factor (HIF) downregulate antigen-presenting MHC class I molecules limiting tumor cell recognition by T cells. PLoS One.

[bib113] Shao J., Hou L., Liu J., Liu Y., Ning J., Zhao Q., Zhang Y. (2021). Indoleamine 2,3-dioxygenase 1 inhibitor-loaded nanosheets enhance CAR-T cell function in esophageal squamous cell carcinoma. Front Immunol.

[bib114] Si X., Shao M., Teng X., Huang Y., Meng Y., Wu L., Wei J., Liu L., Gu T., Song J. (2024). Mitochondrial isocitrate dehydrogenase impedes CAR T cell function by restraining antioxidant metabolism and histone acetylation. Cell Metab.

[bib115] Siegel R.L., Giaquinto A.N., Jemal A. (2024). Cancer statistics, 2024. CA Cancer J Clin.

[bib116] Siegel R.L., Miller K.D., Wagle N.S., Jemal A. (2023). Cancer statistics, 2023. CA Cancer J Clin.

[bib117] Srivastava M.K., Sinha P., Clements V.K., Rodriguez P., Ostrand-Rosenberg S. (2010). Myeloid-derived suppressor cells inhibit T-cell activation by depleting cystine and cysteine. Cancer Res.

[bib118] Srivastava S., Furlan S.N., Jaeger-Ruckstuhl C.A., Sarvothama M., Berger C., Smythe K.S., Garrison S.M., Specht J.M., Lee S.M., Amezquita R.A. (2021). Immunogenic chemotherapy enhances recruitment of CAR-T cells to lung tumors and improves antitumor efficacy when combined with checkpoint blockade. Cancer Cell.

[bib119] Sullivan P.M., Kumar R., Li W., Hoglund V., Wang L., Zhang Y., Shi M., Beak D., Cheuk A., Jensen M.C. (2022). FGFR4-targeted chimeric antigen receptors combined with anti-myeloid polypharmacy effectively treat orthotopic rhabdomyosarcoma. Mol Cancer Ther.

[bib120] Sun T., Liu B., Li Y., Wu J., Cao Y., Yang S., Tan H., Cai L., Zhang S., Qi X. (2023). Oxamate enhances the efficacy of CAR-T therapy against glioblastoma via suppressing ectonucleotidases and CCR8 lactylation. J Exp Clin Cancer Res.

[bib121] Suryadevara C.M., Desai R., Abel M.L., Riccione K.A., Batich K.A., Shen S.H., Chongsathidkiet P., Gedeon P.C., Elsamadicy A.A., Snyder D.J. (2018). Temozolomide lymphodepletion enhances CAR abundance and correlates with antitumor efficacy against established glioblastoma. Oncoimmunology.

[bib122] Tang J., Sheng J., Zhang Q., Ji Y., Wang X., Zhang J., Wu J., Song J., Bai X., Liang T. (2023). Runx3-overexpression cooperates with ex vivo AKT inhibition to generate receptor-engineered T cells with better persistence, tumor-residency, and antitumor ability. J Immunother cancer.

[bib123] Terceiro L.E.L., Ikeogu N.M., Lima M.F., Edechi C.A., Nickel B.E., Fischer G., Leygue E., McManus K.J., Myal Y. (2023). Navigating the blood-brain barrier: challenges and therapeutic strategies in breast cancer brain metastases. Int J Mol Sci.

[bib124] Tettamanti S., Rotiroti M.C., Giordano Attianese G.M.P., Arcangeli S., Zhang R., Banerjee P., Galletti G., McManus S., Mazza M. (2022). Lenalidomide enhances CD23.CAR T cell therapy in chronic lymphocytic leukemia. Leuk Lymphoma.

[bib125] Thieblemont C., Chevret S., Allain V., Di Blasi R., Morin F., Vercellino L., Roulland S., Tarte K., Meignin V., Caillat-Zucman S. (2020). Lenalidomide enhance CAR T-Cells response in patients with refractory/relapsed large B cell lymphoma Experiencing progression after Infusion. Blood.

[bib126] Thornton A.M., Shevach E.M. (1998). CD4+CD25+ immunoregulatory T cells suppress polyclonal T cell activation in vitro by inhibiting interleukin 2 production. J Exp Med.

[bib127] Till B.G., Jensen M.C., Wang J., Qian X., Gopal A.K., Maloney D.G., Lindgren C.G., Lin Y., Pagel J.M., Budde L.E. (2012). CD20-specific adoptive immunotherapy for lymphoma using a chimeric antigen receptor with both CD28 and 4-1BB domains: pilot clinical trial results. Blood.

[bib128] Twomey J.D., Zhang B. (2021). Cancer immunotherapy update: FDA-approved checkpoint inhibitors and companion diagnostics. The AAPS journal.

[bib129] Upton D.H., Ung C., George S.M., Tsoli M., Kavallaris M., Ziegler D.S. (2022). Challenges and opportunities to penetrate the blood-brain barrier for brain cancer therapy. Theranostics.

[bib130] Waldman A.D., Fritz J.M., Lenardo M.J. (2020). A guide to cancer immunotherapy: from T cell basic science to clinical practice. Nat Rev Immunol.

[bib131] Wang D., Yang L., Yue D., Cao L., Li L., Wang D., Ping Y., Shen Z., Zheng Y., Wang L. (2019). Macrophage-derived CCL22 promotes an immunosuppressive tumor microenvironment via IL-8 in malignant pleural effusion. Cancer Lett.

[bib132] Wang X., Rivière I. (2016). Clinical manufacturing of CAR T cells: foundation of a promising therapy. Mol Ther Oncolytics.

[bib133] Wang Y., Tong C., Dai H., Wu Z., Han X., Guo Y., Chen D., Wei J., Ti D., Liu Z. (2021). Low-dose decitabine priming endows CAR T cells with enhanced and persistent antitumour potential via epigenetic reprogramming. Nat Commun.

[bib134] Wang Z., Zhou G., Risu N., Fu J., Zou Y., Tang J., Li L., Liu H., Liu Q., Zhu X. (2020). Lenalidomide enhances CAR-T cell activity against solid tumor cells. Cell Transplant.

[bib135] Watanabe N., Mo F., Zheng R., Ma R., Bray V.C., van Leeuwen D.G., Sritabal-Ramirez J., Hu H., Wang S., Mehta B. (2023). Feasibility and preclinical efficacy of CD7-unedited CD7 CAR T cells for T cell malignancies. Mol Ther.

[bib136] Weber E.W., Lynn R.C., Sotillo E., Lattin J., Xu P., Mackall C.L. (2019). Pharmacologic control of CAR-T cell function using dasatinib. Blood Adv.

[bib137] Weber E.W., Parker K.R., Sotillo E., Lynn R.C., Anbunathan H., Lattin J., Good Z., Belk J.A., Daniel B., Klysz D. (2021). Transient rest restores functionality in exhausted CAR-T cells through epigenetic remodeling. Science.

[bib138] Wei J., Han X., Bo J., Han W. (2019). Target selection for CAR-T therapy. J Hematol Oncol.

[bib139] Wei S.C., Duffy C.R., Allison J.P. (2018). Fundamental mechanisms of immune checkpoint blockade therapy. Cancer Discov.

[bib140] Wing K., Onishi Y., Prieto-Martin P., Yamaguchi T., Miyara M., Fehervari Z., Nomura T., Sakaguchi S. (2008). CTLA-4 control over Foxp3+ regulatory T cell function. Science.

[bib141] Works M., Soni N., Hauskins C., Sierra C., Baturevych A., Jones J.C., Curtis W., Carlson P., Johnstone T.G., Kugler D. (2019). Anti-B-cell maturation antigen chimeric antigen receptor T cell function against multiple myeloma Is Enhanced in the presence of lenalidomide. Mol Cancer Ther.

[bib142] Wulf A.M., Moreno M.M., Paka C., Rampasekova A., Liu K.J. (2021). Defining pathological activities of ALK in neuroblastoma, a neural crest-derived cancer. Int J Mol Sci.

[bib143] Xia L., Liu J.Y., Zheng Z.Z., Chen Y.J., Ding J.C., Hu Y.H., Hu G.S., Xia N.S., Liu W. (2021). BRD4 inhibition boosts the therapeutic effects of epidermal growth factor receptor-targeted chimeric antigen receptor T cells in glioblastoma. Mol Ther.

[bib144] Xia X., Zhou Y., Gao H. (2021). Prodrug strategy for enhanced therapy of central nervous system disease. Chem Commun (Camb).

[bib145] Yan H., Dong M., Liu X., Shen Q., He D., Huang X., Zhang E., Lin X., Chen Q., Guo X. (2019). Multiple myeloma cell-derived IL-32γ increases the immunosuppressive function of macrophages by promoting indoleamine 2,3-dioxygenase (IDO) expression. Cancer Lett.

[bib146] Yan W., Liu X., Ma H., Zhang H., Song X., Gao L., Liang X., Ma C. (2015). Tim-3 fosters HCC development by enhancing TGF-β-mediated alternative activation of macrophages. Gut.

[bib147] Ye H., Zhou Q., Zheng S., Li G., Lin Q., Wei L., Fu Z., Zhang B., Liu Y., Li Z., Chen R. (2018). Tumor-associated macrophages promote progression and the Warburg effect via CCL18/NF-kB/VCAM-1 pathway in pancreatic ductal adenocarcinoma. Cell Death Dis.

[bib148] Yee C. (2014). The use of endogenous T cells for adoptive transfer. Immunol Rev.

[bib149] Yu J., Du W., Yan F., Wang Y., Li H., Cao S., Yu W., Shen C., Liu J., Ren X. (2013). Myeloid-derived suppressor cells suppress antitumor immune responses through IDO expression and correlate with lymph node metastasis in patients with breast cancer. J Immunol.

[bib150] Zarei M., Abdoli S., Farazmandfar T., Shahbazi M. (2023). Lenalidomide improves NKG2D-based CAR-T cell activity against colorectal cancer cells invitro. Heliyon.

[bib151] Zhang C., Liu J., Zhong J.F., Zhang X. (2017). Engineering CAR-T cells. Biomark Res.

[bib152] Zhang F., Stephan S.B., Ene C.I., Smith T.T., Holland E.C., Stephan M.T. (2018). Nanoparticles that reshape the tumor milieu create a therapeutic window for effective T-cell therapy in solid malignancies. Cancer Res.

[bib153] Zhang L., Jin G., Chen Z., Yu C., Li Y., Li Y., Chen J., Yu L. (2021). Lenalidomide improves the antitumor activity of CAR-T cells directed toward the intracellular Wilms Tumor 1 antigen. Hematology.

[bib154] Zhang Q., Zhang H., Ding J., Liu H., Li H., Li H., Lu M., Miao Y., Li L., Zheng J. (2018). Combination therapy with EpCAM-CAR-NK-92 cells and regorafenib against human colorectal cancer models. J Immunol Res.

[bib155] Zhang W., Shi L., Zhao Z., Du P., Ye X., Li D., Cai Z., Han J., Cai J. (2019). Disruption of CTLA-4 expression on peripheral blood CD8 + T cell enhances anti-tumor efficacy in bladder cancer. Cancer Chemother Pharmacol.

[bib156] Zhang X., Sun S., Miao Y., Yuan Y., Zhao W., Li H., Wei X., Huang C., Hu X., Wang B. (2022). Docetaxel enhances the therapeutic efficacy of PSMA-specific CAR-T cells against prostate cancer models by suppressing MDSCs. J Cancer Res Clin Oncol.

[bib157] Zhang Y., Zhang X., Cheng C., Mu W., Liu X., Li N., Wei X., Liu X., Xia C., Wang H. (2017). CRISPR-Cas9 mediated LAG-3 disruption in CAR-T cells. Front Med.

[bib158] Zhang Z., Wang G., Zhong K., Chen Y., Yang N., Lu Q., Yuan B., Wang Z., Li H., Guo L. (2023). A drug screening to identify novel combinatorial strategies for boosting cancer immunotherapy efficacy. J Transl Med.

[bib159] Zhao P., Jiang D., Huang Y., Chen C. (2021). EphA2: A promising therapeutic target in breast cancer. J Genet Genomics.

[bib160] Zhao S., Li J., Xia Q., Liu K., Dong Z. (2023). New perspectives for targeting therapy in ALK-positive human cancers. Oncogene.

[bib161] Zhao Z., Condomines M., van der Stegen S.J.C., Perna F., Kloss C.C., Gunset G., Plotkin J., Sadelain M. (2015). Structural design of engineered costimulation determines tumor rejection kinetics and persistence of CAR T cells. Cancer Cell.

[bib162] Zhong M., Gao R., Zhao R., Huang Y., Chen C., Li K., Yu X., Nie D., Chen Z., Liu X. (2022). BET bromodomain inhibition rescues PD-1-mediated T-cell exhaustion in acute myeloid leukemia. Cell Death Dis.

[bib163] Zhu X., Li W., Gao J., Shen J., Xu Y., Zhang C., Qian C. (2023). RUNX3 improves CAR-T cell phenotype and reduces cytokine release while maintaining CAR-T function. Med Oncol.

