# Supplementary material for: Therapeutic advantage of combinatorial chimeric antigen receptor T cell and chemotherapies
Source: Pharmacol Rev. 2024 Nov 22;77(1):100011. doi: 10.1124/pharmrev.124.001070 (PMC13095475; doi:10.1124/pharmrev.124.001070)
Supplement: Supplementary Table 1 [file mmc1.pdf]

Therapeutic advantage of combinatorial CAR T cell and chemo-therapies

Meghan B. Ward*, Amber B. Jones*, Giedre Krenciute

Pharmacological Reviwes:
MS ID#: PHARMREV-AR-2023-001070R1

Supplementary Table 1: Expanded list of preclinical CAR T and chemotherapy studies including relevant mechanisms of action

Drug (s)		FDA Appr. (Y/N)	Drug Description	CAR Target (s)	Tumor Model (s)	CAR Limitation (s) Addressed	Relevant Biological Mechanism(s)	PMID
**1**	All trans retanoic acid (tretinoin)	Yes	Vitamin A Derived Retinoid	BCMA	Multiple Myeloma	Antigen Dilema	Enhanced antigen expression on primary myeloma cells	36722406
**2**	All trans retanoic acid (tretinoin)	Yes	Vitamin A Derived Retinoid	CD38	Multiple Myeloma	Antigen Dilema	Upregulated antigen expression on tumor cells which potentiated CAR T cell killing	36918219
**3**	Azacitidine	Yes	Hypomehylating Agent	CD70	Acute Myeloid Leukemia	Antigen Dilema	Increased antigen expression on tumor cells in a high tumor burden bearing model	35452603
**4**	Bryostatin-1	Yes	modulate Protein Kinase C	CD22	Leukemias and lymphomas	Antigen Dilema	Upregulated CD22 on different leukemia and lymphoma cells which improved CAR T cell functionality and in vivo persistence	31110075
**5**	Bryostatin-1	Yes	modulate Protein Kinase C	CD22	B-ALL	Antigen Dilema	Increased cytolysis through enhanced antingen expression on cancer cell lines while sparing normal cells	35222407
**6**	Cisplatin	Yes	Alkylating Agent	CD133	Gastric Cancer	Antigen Dilema	Pretreament of tumor cells iwith drug mproved tumor cell killing by CAR T cells	33635343
**7**	Crenolanib	Yes	Kinase Inhibitor	FLT3	Acute Myeloid Leukemia	Antigen Dilema	Upregulated antigen expression leading to increased CAR T cell mediated killing	29472720
**8**	Cyclophos-phamide	Yes	Lymphodepleting Agent	NKG2D ligands	Off-Target Study Only	Antigen Dilema	Upregulated NKG2D ligands which improved cytotoxicity of CAR T cells	26122933

**9**	Decitabine	Yes	Hypomehylating Agent	CSPG4	Ovarian Cancer	Antigen Dilema	Mediated a dose dependent increase in CSPG4 on antigen negative cells enhancing recognition by CAR T cells	36291817
**10**	Decitabine	Yes	Hypomehylating Agent	CD19	Lymphoblastoma	Antigen Dilema	Increased antigen expression on tumor cells improving CAR T cell mediated killing	31372000
**11**	Decitabine and Chidamide	Yes	Hypomehylating Agent and HDAC Inhibitor	nanobodyCD70	Acute Myeloid Leukemia	Antigen Dilema	Combinatorial treament enhanced the expression of CD70 on AML cells which facilitated greater cytotoxic activity of CAR T cell	36932256
**12**	Gemcitabine	Yes	Antimetabolite	GRP78	Pancreatic Cancer	Antigen Dilema	Increased surface antigen expression leading to increased tumor killing by CAR T cells	37897831
**13**	Ingenol-3-angelate	Yes	Protein Kinase C Agonist	B7-H3	Osteosarcoma	Antigen Dilema	Increased surface expression of B7-H3 via PKC activation	38561833
**14**	Lenalidomide	Yes	Immunomodulatory Agent	MUC1	Multiple Myeloma	Antigen Dilema	Increased antigen expression on tumor cells improving CAR T cell mediated killing	35840578
**15**	Lorlatinib	Yes	Broad Kinase Inhibitor	CD19, GD2, ALK	Leukemia, Neuroblastoma	Antigen Dilema	Increased antigen expression on low antigen density cells potentiating CAR T cell effector function	38039964
**16**	ABT-737	No	Bcl-2 Inhibitor	CD19	B Cell Malignancy	Tumor Burden	Increased tumor cell apoptosis	23788110
**17**	Azacitidine	Yes	Hypomethylating Agent	CEA	Colorectal Cancer	Tumor Burden	Increased antigen expression on tumor cells and improved cytotoxic effect of CAR T cells which may be of resistant to other chemotherapy resistant models	30075754
**18**	Celecoxib	Yes	COX2 Inhibitor	CD19	B Cell Malignancy	Tumor Burden	Partially reversed CAR T cell resistance of tumor cell lines	29904021

**19**	Dabrafenib	Yes	MAPK Inhibitor	GD2	Melanoma	Tumor Burden	Used in the clinic to treat melanoma, need to see if can be used in combination but little impact on CAR T cell function individually	25415284
**20**	Decitabine	Yes	Hypomehylating Agent	EGFR, CD44v6	Bladder cancer	Tumor Burden	Differential regulated the expression of cell survial and apoptosis factors skewing the balance towards more apoptosis sensitivity which enhanced CAR T cell killing	34868059
**21**	Fluorouracil	Yes	Anti-Metabolite	CEA	Colorectal Cancer	Tumor Burden	Increased antigen expression on tumor cells but negatively influenced the viability of the CAR T cells	30075754
**22**	Indometacin	Yes	NSAID	CD19	B Cell Lymphoma	Tumor Burden	Sensitized tumor cells to CAR T cells by inducing oxidative stress which led to increased death receptor surface expression	35882449
**23**	Paclitaxel	Yes	Anti-Microtubule Agent	T4	Epithelial Ovarian Cancer	Tumor Burden	Induced tumor cell apopotosis and promoted cell cycle arrest offerign additive benefit to CAR T cell therapy	30167862
**24**	Rimiducid	Yes	Dimerizing Agent	IL-1RAP	Acute Myeloid Leukemia	Tumor Burden	Eliminated tumor cells that were resistant to CAR T cell therapy whose resistance was driven by epitope masking	33414517
**25**	Sodium Butyrate	No	HDAC Inhibitor	CEA	Colorectal Cancer	Tumor Burden	Increased antigen expression on tumor cells and improved cytotoxic effect of CAR T cells which may be of resistant to other chemotherapy resistant models	30075754
**26**	THZ1	No	Broad Kinase Inhibitor	EGFR	Triple Negative Breast Cancer	Tumor Burden	Overcame tumor acquired CAR T cell resistance thorugh transcriptional reprogramming preventing further resistnace, tumor growth, and metastasis	33875483

**27**	Trametinib	Yes	MEK Inhibitor	GD2	Melanoma	Tumor Burden	Used in the clinic to treat melanoma, need to see if can be used in combination but little impact on CAR T cell function individually	25415284
**28**	Vemurafenib	Yes	MAPK Inhibitor	GD2	Melanoma	Tumor Burden	Used in the clinic to treat melanoma, need to see if can be used in combination but compound did impair CAR T cells at plasma relevant concentrations	25415284
**29**	Zanubrutinib	Yes	Src Kinase Inhibitor	CD19	Lymphoblasto ma	Tumor Burden	Induced dose dependent toxitcty on tumor cells and CAR T cells	36254554
**30**	DMXAA	No	STING Agonist	Neu	Breast Cancer	Infiltration	Improved infiltrration of raditional Th1 type CAR T cells but more significnalty synergized with Th/Tc17 CAR T cell phenotypes	33382402
**31**	Docetaxel	Yes	Anti-Neoplastic Agent	HER-2	Non-Small Cell Lung Cancer	Infiltration	Increased the recruitment and infiltrative capacity of CAR T cells through a potential CXCL11 mechanism	30744691
**32**	Rapamycin	Yes	mTOR Inhbitor	EpCAM	Acute Myeloid Leukemia	Infiltration	Improved the capacity of CAR T cells to infiltrate into the bone marrow by modulating mTOR target CXCR4	34233960
**33**	All trans retanoic acid (tretinoin)	Yes	Vitamin A Derived Retinoid	FGFR4	Rhabdomyosar coma	Immune Microenvironment	Targets myeloid derived suppressor cells	35877472
**34**	BLZ945	No	CSF1R Inhibitor	B7-H3	Glioma	Immune Microenvironment	Depleted tumor associated macrophages but was actually detrimental to CAR T cell efficacy	37971169
**35**	Carboplatin	Yes	Alkylating Agent	Lewis Y antigen (LeY)	Prosate cancer	Immune Microenvironment	Improved the efficacy of CAR T cell treatment but the response was dependent on the changes induced by the tumor microenvironment	37660083

**36**	Cyclophos-phamide	Yes	Lymphodepleting Agent	CD19	Raji tumors	Immune Microenvironment	Depleted tumor associated Tregs	21487038
**37**	Cyclophos-phamide	Yes	Lymphodepleting Agent	CD19	Raji tumors	Immune Microenvironment	Contributed to tumor clearance in combination with CAR T cells	18477047
**38**	Cyclophos-phamide	Yes	Lymphodepleting Agent	CEA	Colon Cancer, Breast Cancer	Immune Microenvironment	Aided in tumor clearance of initially established tumors and helped induce tumor immunity in re-challenged settings	33796409
**39**	Cyclophos-phamide	Yes	Lymphodepleting Agent	PSCA	Prostate, Pancreatic	Immune Microenvironment	Dampened immunosuppressive microenvironment and supported pro-inflammatory microenvironment	33647456
**40**	Cyclophos-phamide and Fludarabine	Yes	Lymphodepleting Agent	CD19	B Cell Leukemia	Immune Microenvironment	Downregulated IDO expression	25940712
**41**	Cyclophos-phamide and Fludarabine	Yes	Lymphodepleting Agent	B7-H3	Canine Sarcoma	Immune Microenvironment	Demonstrated efficacy of CAR T cells in canine sarcoma with cyclophosphamide and fludaribine successfully used as lymphopdepleting agents	35405743
**42**	Docetaxel	Yes	Anti-Neoplastic Agent	PSMA	Prostate Cancer	Immune Microenvironment	Improved cytotoxic effect of CAR T cells by diminishing the frequency and immunosuppressive capacity of MDSCs	35962287
**43**	Epacadostat	Yes	IDO1 Inhibitor	FGFR4	Rhabdomyosarcoma	Immune Microenvironment	Promtoted the successsful clearance of RMS tumors using a polypharmacy approach	35877472
**44**	Epacadostat	Yes	IDO-1 Inhibitor	MSLN	Esophageal Squamous Cell Carcinoma	Immune Microenvironment	Prevented the accumulation of the immunosuppresive metabolite,kynurenine, which enhanced cytotoxic potential	33828565
**45**	L-NAME	No	iNOS Inhibitor	FGFR4	Rhabdomyosarcoma	Immune Microenvironment	Promtoted the successsful clearance of RMS tumors using a polypharmacy approach	35877472

**46**	Oxamate	No	Lactate Dehydrogenase A Inhbitor	EGFRvIII	Glioblastoma	Immune Microenvironment	Modulated the immunosuppressive microenvironment by reducing promoter activity of Treg populations and restraining chemokine activity in macrophages	37770937
**47**	Pexidartinib	Yes	CSF1R Inhibitor	FGFR4	Rhabdomyosar coma	Immune Microenvironment	targets M2 like TAMs	35877472
**48**	PI-3065	No	PI3K Inhibitor	ROR1, EGFRvIII	Breast	Immune Microenvironment	Nanoparticle loaded sheets improved CAR T cell effector function through remodeling of the immune microenvironment	29760047
**49**	SD-208	No	TGFβ Inhibitor	FGFR4	Rhabdomyosar coma	Immune Microenvironment	Protected CAR T cells from inhibitory effect of TGF-β and reduced PD-1 expression within a polypharmacy approach	36722406
**50**	SD-208	No	TGFβ Inhibitor	ROR1	Triple-Negative Breast Cancer	Immune Microenvironment	Protected CAR T cells from inhibitory effect of TGF-β and reduced PD-1 expression within a polypharmacy approach	32303620
**51**	7DW8-5	No	Immunostimulatory Glycolipid	ROR1, EGFRvIII	Breast	Immune Microenvironment	Nanoparticle loaded sheets improved CAR T cell effector function through remodeling of the immune microenvironment	29760047
**52**	Acalabrutinib	Yes	Src Kinase Inhibitor	CD19	Lymphoblasto ma	Fitness	Improved in vitro persistence and in vivo tumor clearance,	31899702
**53**	AKT Inhbitor VIII	No	PI3K Inhibitor	CD19	B cell leukemia	Fitness	Minimized differentiation of CAR T cells through induced regulation of the FOXO1 transcription factor	29212954
**54**	Carboplatin	Yes	Alkylating Agent	ErbB	Epithelial ovarian cancer	Fitness	Tumor pre-treatment with noncytotoxic doses of carboplatin sensitized EOC tumors to CAR T cells resulting in better disease regression while using lower doses of CAR T cells	23898037

**55**	Carboplatin	Yes	Alkylating Agent	EGFR	Breast cancer (TNBC; MDA-MDB-468 cells)	Fitness	Improved CAR T cell cytotoxic activity in vitro	35813488
**56**	Celecoxib and aspirin	Yes	COX1/2 Inhibitors	CD19	B Cell Lymphoma	Fitness	COX inhibition induced tumor cell apoptosis but impaired quality of CAR T cells	34122428
**57**	Dasatinib	Yes	Src Kinase Inhibitor	GD2	B Lymphoid Leukemia	Fitness	Promoted memory phenotype, decreased exhaustion markers, and increased effector cytokine production	33795428
**58**	Dasatinib	Yes	Src Kinase Inhibitor	GRP78	Acute Myeloid Leukemia	Fitness	Prevented CAR T cell antigen mediated differentation which improved cytotoxicity	35102167
**59**	Dasatinib	Yes	Src Kinase Inhibitor	CD19	B Lymphoid Leukemia	Fitness	Served as a safety switch for CAR T cell by limiting cytotoxicity, cytokine secretion, and proliferation	30814055
**60**	Dasatinib	Yes	Src Kinase Inhibitor	CD19	Lymphoblastoma	Fitness	Paused CAR T cell intracellullar signaling transduction by interefering with upstream kinase activity	31270272
**61**	Dasatinib	Yes	Src Kinase Inhibitor	CD19	B Lymphoid Leukemia	Fitness	Decreased exhaustion, differentiation and apoptosi related gene signatures	34289897
**62**	Dasatinib and Ibrutinib	Yes	Src Kinase Inhibitor	CD7	T cell Leukemia	Fitness	Minimized tonic signaling induced fratricide on the CAR T cell and improved persistance and tumor control	36086817
**63**	Decitabine	Yes	Hypomehylating Agent	CD19	Lymphoblastoma	Fitness	Enhanced memory phenotype, proliferation, and cytokine production of CAR T cells while also mitigating exhaustion	33462245

**64**	Decitabine	Yes	Hypomehylating Agent	CD123	Leukemia	Fitness	Epigenetically reprogammed CAR T cells which decreased methyltransferase expression leading to decreased exhaustion markers and ehnanced memory phenotype	32973749
**65**	Dexamethasone	Yes	Anti-Inflammatory Synthetic Glucocorticoid	IL13Ra2	Glioblastoma	Fitness	Identified threshold that which dexamethasone is harmful for CAR T cells	35081104
**66**	Dexamethasone	Yes	Anti-Inflammatory Synthetic Glucocorticoid	IL13Ra2	Glioblastoma	Fitness	Dex is commonly used in GBM tretament, low doses do not impact CAR T cell functionality - but do not necessarially help either	29103912
**67**	Dexamethasone	Yes	Anti-Inflammatory Synthetic Glucocorticoid	CD19, CS-1, TAG-72	ALL, Multiple Myeloma, Ovarian Cancer	Fitness	Dex increases IL-7 receptor expression on CAR T cells, further combination with IL-7 enhanced anti-tumor activity	38140726
**68**	Dexamethasone and methylpred-nisone	Yes	Anti-Inflammatory Synthetic Glucocorticoid	CD19, MSLN	Leukemia	Fitness	Exposure to GCs impairs CAR T cell activity but is reversed when GCs are removed, Important when GCs are used to control CRS but cytotoxicity is still desired	38475830
**69**	Docetaxel	Yes	Anti-Neoplastic Agent	PSMA	Prostate Cancer	Fitness	Combinatorial treatment proved to have additive effect at killing antigen positive tumor cells	32728611
**70**	Enasidenib	Yes	IDH2 Inhibitor	CD19	Leukemia, Osteosarcoma	Fitness	Enhances persistence, expansion, and memory CAR T cell formation through enhanced glycolytic activity and reduced reacitve oxygen species formation	38171332
**71**	Ibrutinib	Yes	Src Kinase Inhibitor	CD19	Chronic Lymphocytic Leukemia	Fitness	Increased CAR T cell viability and expansion while decreasing differentiation and exhaustion markers and imporving cytokine release	32683672

**72**	Ibrutinib	Yes	Src Kinase Inhibitor	CD19	Lymphoblasto ma	Fitness	Decreased exhaustion marker and displayed better combined tumor control when added to CAR T cells	32876369
**73**	Ibrutinib	Yes	Src Kinase Inhibitor	CD19	Mantle Cell Lymphoma	Fitness	Enhanced CAR T cell killing and improved long term remission of mice and exhaustion markers in vivo	26819453
**74**	Ibrutinib	Yes	Src Kinase Inhibitor	CD19	Chronic Lymphocytic Leukemia	Fitness	Improves CAR T cell engraftment supporting greater tumor clearence	26813675
**75**	Ibrutinib	Yes	Src Kinase Inhibitor	CD19	Lymphoblasto ma	Fitness	Improved in vitro persistence, in vivo tumor clearance,and promoted a memory phenotype.	31899702
**76**	Idelaslisib	Yes	PI3K Inhibitor	CD19	Chronic Lymphocytic Leukemia	Fitness	Enriched the naive T CAR T cell fraction, increased lymph node homing markers, and decreased exhaustion marker expression	30737788
**77**	IPI-145,CAL-101, or TGR-1202	No	PI3K Inhibitor	MSLN	Melanoma	Fitness	Dual blockade of the gamma and delta subunits of PI3K impaired cell function but individual use with either inhibitor improved anti-tumor function	32383488
**78**	JQ1	No	BET Bromodomain Inhibitor	EGFR	Glioblastoma	Fitness	Suppressed the transcriptional expression of CAR T cell induced immunosuppressive genes supporting cytotoxic argeting of tumor cells	34058385
**79**	JQ1	No	BET Bromodomain Inhibitor	CD19	Chronic Lymphocytic Leukemia	Fitness	Reinvigorated exhausted CAR T cells through increased CAR expansion, proliferation, and metabolic fitness	34396987
**80**	Lenalidomide	Yes	Immunomodulatory Agent	CD133, HER2	Glioblastoma, Breast Cancer	Fitness	Increased CAR T effector function (prolerfation, cytokine secretion,and killing) through drug regulated degradation of T cell essential transcription factors	32967454

**81**	Lenalidomide	Yes	Immunomodulatory Agent	CD19, BCMA	Lymphoblastoma	Fitness	Drug regulated degradable IL-7 system that enhanced CAR T cell expansion, memory status, and persistence and antitumor activity of CAR T cells	38123696
**82**	Lenalidomide	Yes	Immunomodulatory Agent	NKG2D	Colorectal Cancer	Fitness	Improved in vitro cytotoxicity and effector cytokine production	37790973
**83**	Lenalidomide	Yes	Immunomodulatory Agent	CD23	Chronic Lymphocytic Leukemia	Fitness	Improved CAR T cell migration and preserved the functionality of CAR T cell immune synapse improving effector function	35259043
**84**	Lenalidomide	Yes	Immunomodulatory Agent	CD19	Lymphoblastoma	Fitness	Served as an "ON" and "OFF"supporting the activation or degradation of CAR T cells which improved antitumor activity while limiting inflammatory cytokine release	33408186
**85**	Lenalidomide	Yes	Immunomodulatory Agent	CD19	Lympoblastic Leukemia	Fitness	Controlled effector function of degron tagged CAR T cells to better regulate activity and minimize off-target toxicitiies	33333026
**86**	Lenalidomide	Yes	Immunomodulatory Agent	BCMA	Multiple Myeloma	Fitness	Enhanced cytokine production, cytolytic activity, activation, and decreased exhaustion markers improving in vivo tumor clearance	31395689
**87**	Lenalidomide	Yes	Immunomodulatory Agent	CS1	Multiple Myeloma	Fitness	Supported increased cytotoxicity and persistence in vivo with improved memory phenotype, Th1 cytokine production,and immune synapse formation	29061640
**88**	LY294002	No	PI3K Inhibitor	NKG2D	Breast Cancer, Lung Cancer	Fitness	Supported CAR T cell survival and prevented exhaustion by upregulating anti-apoptotic proteins	35965586
**89**	LY294002	No	PI3K Inhibitor	NKG2D	Chronic Myeloid Leukemia, Pancreatic Cancer	Fitness	Decreased antigen expression on activated CAR T cells diminishing fratricididic cell death	30619300

**90**	LY294002	No	PI3K Inhibitor	CD33	Acute Myeloid Leukemia	Fitness	Improved persisitence by mediating a less differentiated state and decreasing tonic CAR T cell signaling	29479065
**91**	Metformin	Yes	Antihyperglycemic	CD19	Lymphoma	Fitness	Suppressed the AMPK pathway which negatively influenced proliferation and cytotoxicity of CAR T cells	29662316
**92**	Paclitaxel	Yes	Anti-Microtubule Agent	ICAM-1	Gastric Cancer	Fitness	Enhanced anti-tumor activity of CAR T cells in vivo	32995483
**93**	Rapamycin	Yes	mTOR Inhibitor	CD123,HER2, CD33	Acute Myeloid Leukemia	Fitness	Used as a pro-apoptotic compound that allowed for in vivo clearance control of CAR cells to better mitigate off target toxicities	32384544
**94**	Rapamycin	Yes	mTOR Inhbitor	BCMA,CD123 (Natural Killer Cells)	Acute Myeloid Leukemia	Fitness	Used as a safety switch to regulate the elimination of expanded effector cells	32384544
**95**	Rapamycin	Yes	mTOR Inhbitor	IL-1RAP	Acute Myeloid Leukemia	Fitness	Used as a safety switch to regulate the elimination of expanded effector cells	37173386
**96**	Rapamycin	Yes	mTOR Inhbitor	CD19,BCMA	Lymphoma	Fitness	Allowed for selective activation of CAR T cells only in the presence of both the antigen and the dimerizing compound	31039141
**97**	Rapamycin	Yes	mTOR Inhbitor	CD19	Lymphoblasto ma	Fitness	Promoted a less differentiated, stem cell like memory phenotype	30890531
**98**	Rapamycin	Yes	mTOR Inhbitor	CD19	Lymphoblasto ma	Fitness	Utilized rapamycin as a caspase regulating compound to initiate CAR T cell suicide and minimize toxicity	29661681
**99**	Regorafenib	Yes	Broad Kinase Inhibitor	EpCAM (Natural Killer Cell)	Colorectal Cancer	Fitness	Supported greated in vivo tumor control through enhanced persistence	30410941
**100**	Rimiducid	Yes	Dimerizing Agent	SLAMF7	Multiple Myeloma	Fitness	Performed as a suicide switch on CAR T cells to better control off target toxicities to antigen containing leukocytes	30740516

**101**	Rimiducid	Yes	Dimerizing Agent	CD123,HER2, CD33	Acute Myeloid Leukemia	Fitness	Used as a pro-stimulatory compound that enhanced CAR T cell proliferation, cytokine secretion, and cytotoxicity	30740516
**102**	Ruxolitinib	Yes	Janus Kinase Inhibitor	CD19	Lymphoblasto ma	Fitness	Inhibited proliferation without damaging viability or effector function of the CAR T cells which helped mitigate cytokine release syndrome related toxicities	35101664
**103**	SCH-58261	No	A2α Receptor Inhibitor	CD19	Leukemia	Fitness	CAR T cells were loaded with vessicles carrying SCH supporting higher CAR T cell engraftment and enhanced functionality	29720380
**104**	SCH-58261	No	A2α Receptor Inhibitor	MSLN	Ovarian Adenocarcino ma	Fitness	Adenosine signling was exogenously activated and co-incubation with SCH reversed loss of CAR T cell effector function and cytokine secretion	32151275
**105**	Temozolomide	Yes	Alkylating Agent	EGFRvIII	Glioblastoma	Fitness	Served as a lymphodepleting agent prior to CAR delivery which improved the abundance and persistence of cells	29872570
**106**	THZ1	No	Broad Kinase Inhibitor	CD19	Lymphoma	Fitness	Protecte against cytokine release syndrome assocaited toxicities by diminishing polymerase medaited transciriptional activity	33397398
**107**	Trametinib	Yes	MEK Inhibitor	GD2	Neuroblastoma	Fitness	Enhanced in vitro tumor cell killing while sparing exhaustion and promoting expansion	34382720
**108**	Azacitidine	Yes	Hypomehylating Agent	CD123	Acute Myeloid Leukemia	Antigen Dilemma, Fitness	Upregulated antigen expression and induced clonal outgrowth of exhuastion marker negative, CAR positive T cells that had increased killing potency	34750374

**109**	Decitabine	Yes	Hypomehylating Agent	NY-ESO-1	Breast Cancer, Multiple Myeloma	Antigen Dilemma, Fitness	Upregulated antigen expression on tumor cells leading to increased killing which also correlated with higher interferon gamma production	26447882
**110**	S63845	No	Mcl-1 Inhibitor	CD19	B-cell Malignancies	Antigen Dilemma/ Tumor Burden	Increased CD19 expression and induced tumor cell apoptosis	33362794
**111**	Venetoclax	yes	Bcl-2 Inhibitor	CD19	B-cell Malignancies	Antigen Dilemma/ Tumor Burden	Increased CD19 expression and induced tumor cell apoptosis where one out of three delivery methods tested was able to prevent CAR T cell damage	33362794
**112**	Venetoclax	yes	Bcl-2 Inhibitor	CD19	Multiple Lymphoma Models	Antigen Dilemma/ Tumor Burden	Overexpressed Bcl2(F104L) to protect CAR T cellf from venetoclax toxicities	35904479
**113**	Cisplatin	Yes	Alkylating Agent	HER2	Lung Cancer	Fitness, Infiltration	Cisplatin-loaded CAR T cells (via membrane coated polymeric nanoparticles) resulted in better anti-tumor response and homing.	38282968
**114**	Lenalidomide	Yes	Immunomodulatory Agent	CD19	B Cell Lymphoma	Fitness, Infiltration	Polarized CD8+ cells to less differentiated state, increased expansion, suppressed VEGF production supporting infiltration, and minimized exhaustion	37219767
**115**	Lenalidomide	Yes	Immunomodulatory Agent	Wilms Tumor 1	Chronic Myelogenous Leukemia	Fitness, Infiltration	Increased infiltration of both CD3/8+ T cells and improves anti-tumor activity	34674611
**116**	Lenalidomide	Yes	Immunomodulatory Agent	CD19,CD20	B-cell non-Hodgkin Lymphoma	Fitness, Infiltration	Increased interferon gamma production and infiltration of CAR positive cells which supported greater anti-tumor activity	27141398
**117**	Lenalidomide	Yes	Immunomodulatory Agent	EGFRvIII	Glioblastoma	Fitness, Infiltration	Improved interferon gamma production, migration of CAR T cells to tumor site, and enhanced immune synapse formation improving tumor control	26450624

**118**	Sorafenib	Yes	Broad Kinase Inhibitor	GPC3	Hepatocellular Cancer	Immune Microenvironment, Tumor Burden	Promtoed IL-12 secretion in tumor associated macrophages and induced tumor cell apoptosis	31078430
**119**	Oxaliplatin (with cyclophos-phamide)	Yes	Alkylating Agent	ROR1	Lung Cancer	Infiltration, Immune Microenvironment	Improves CAR-T cell-mediated tumor control and survival when combined with cyclophosphamide by activating tumor macrophages to express T-cell-recruiting chemokines, resulting in improved CAR-T cell infiltration, remodeling of the tumor microenvironment, and increased tumor sensitivity to anti-PD-L1	33357452
**120**	Azacitidine	Yes	Hypomehylating Agent	CD44v6	Acute Myeloid Leukemia	Tumor Burden, Fitness	Increased the activation, persistence, and promoted a memory phenotype of CAR T cells while also inducing tumor celll apoptosis	37180104
**121**	Decitabine	Yes	Hypomehylating Agent	CD44v6	Acute Myeloid Leukemia	Tumor Burden, Fitness	Increased the activation, persistence, and promoted a memory phenotype of CAR T cells while also inducing tumor celll apoptosis (more profound than azacitidine)	37180104
**122**	Eltanexor	No	XPO-1 Inhibitor	CD19	Lymphoma, Acute Myeloid Leukemia	Tumor Burden, Fitness	Pretreament of tumor cells with inhibitor sensistized them to CAR T cell therapy by decreasing tumor cell viability and mitigating exhaustion	34165175
**123**	Ibrutinib	Yes	Src Kinase Inhibitor	CD19	Lymphoblastoma	Tumor Burden, Fitness	Induced dose dependent toxiticty on tumor cells and CAR T cells but decreased exhaustion markers and had better anti-tumor effect	36254554

**124**	JQ1	No	BET Bromodomain Inhibitor	CD19,CD123	Acute Myeloid Leukemia	Tumor Burden, Fitness	Decreased immunosuppressive ligand on tumor cells and their respective receptors on CAR T cells supporting anti-tumor activity	36038554
**125**	Linsitinib	Yes	IGF1R/IR Inhibitor	GD2	Diffuse Midline Glioma	Tumor Burden, Fitness	Induced tumor cell apoptosis while not affecting CAR T cell viability and increased memory phenotype expression as well as decreased exhaustion markers	34964902
**126**	Metformin	Yes	Antihyperglycemic	CEA	Gastric Cancer	Tumor Burden, Fitness	Blocked tumor specific aerobic glycolysis while upregulating CAR T cell oxidative phosphorylation and energy metabolism	36827893
**127**	Rapamycin	Yes	mTOR Inhbitor	CD19	B cell Lymphoma	Tumor Buden Fitness,	Inhibited tumor specific immune evasion and anti-apoptic mechanisms while working in tandem withengineered rapamycin resistant CAR T cells	21878902
**128**	Selinexor	Yes	XPO-1 Inhibitor	CD19	Lymphoma, Acute Myeloid Leukemia	Tumor Burden, Fitness	Pretreament of tumor cells with inhibitor sensistized them to CAR T cell therapy by decreasing tumor cell viability and mitigating exhaustion	34165175
**129**	JK184	No	Hedghog Inhbibitor	B7-H3	Breast Cancer	Tumor Burden, Infiltration	Promoted tumor cell apoptosis and CAR T cell traficking and infiltration to the tumor site which corresponded to increased inflammatory cytokine production	36635683
**130**	Sunitinib	Yes	Broad Kinase Inhibitor	CAIX	Renal Cancer	Antigen Dilemma, Infiltration, Immune Microenvironment	Upregulated antigen expression on tumor cells, promoted CAR T cell infiltration, and reduced the abundance and frequency of myeloid derived suppressor cells	31574023

**131**	Metformin and Rapamcyin	Yes	AMPK Activator, mTOR Inhibitor	EGFRvIII	Glioblastoma	FItness, Immune Microenvironment, Tumor Burden	Pretreated CAR T cells had better persistence and anti-tumor activity under impairing hypoxic conditions along with fewer MDSCs	38386420
**132**	TY-52156	No	S1P3 Receptor Antagonist	EpCAM	Breast Cancer, Colon Cancer	Fitness, Infiltration, Immune Microenvironment	Improved infiltratiion and effector function by remodeling the TME to promote pro-inflammatory macrophage polarization and recruitment	37591632

